# Bursting of excitatory cells is linked to interictal epileptic discharge generation in humans

**DOI:** 10.1038/s41598-022-10319-4

**Published:** 2022-04-15

**Authors:** Katharina T. Hofer, Ágnes Kandrács, Kinga Tóth, Boglárka Hajnal, Virág Bokodi, Estilla Zsófia Tóth, Loránd Erőss, László Entz, Attila G. Bagó, Dániel Fabó, István Ulbert, Lucia Wittner

**Affiliations:** 1 Institute of Cognitive Neuroscience and Psychology, Research Center for Natural Sciences, Eötvös Loránd Research Network, Magyar tudósok körútja 2., 1117 Budapest, Hungary; 2 Faculty of Information Technology and Bionics, Pázmány Péter Catholic University, 1083 Budapest, Hungary; 3 National Institute of Mental Health, Neurology and Neurosurgery, 1143 Budapest, Hungary; 4 Semmelweis University Doctoral School, 1026 Budapest, Hungary; 5 Present Address: Department of Neurobiology, School of Medicine and Institute for Medical Research Israel-Canada, The Hebrew University, 91120 Jerusalem, Israel

**Keywords:** Diseases of the nervous system, Epilepsy, Neural circuits, Neuroscience, Excitability, Inhibition-excitation balance

## Abstract

Knowledge about the activity of single neurons is essential in understanding the mechanisms of synchrony generation, and particularly interesting if related to pathological conditions. The generation of interictal spikes—the hypersynchronous events between seizures—is linked to hyperexcitability and to bursting behaviour of neurons in animal models. To explore its cellular mechanisms in humans we investigated the activity of clustered single neurons in a human in vitro model generating both physiological and epileptiform synchronous events. We show that non-epileptic synchronous events resulted from the finely balanced firing of excitatory and inhibitory cells, which was shifted towards an enhanced excitability in epileptic tissue. In contrast, interictal-like spikes were characterised by an asymmetric overall neuronal discharge initiated by excitatory neurons with the presumptive leading role of bursting pyramidal cells, and possibly terminated by inhibitory interneurons. We found that the overall burstiness of human neocortical neurons is not necessarily related to epilepsy, but the bursting behaviour of excitatory cells comprising both intrinsic and synaptically driven bursting is clearly linked to the generation of epileptiform synchrony.

## Introduction

Information about the activity of neocortical single cells is very important in understanding how physiological and pathological synchronisations are generated in the brain. Large amounts of data are available about the cellular mechanisms of normal and epileptic oscillatory activity in the rodent brain (for review see^1^). Human data are sparse, although especially valuable to get insight into the working mechanisms and the pathologies of the human brain. In addition, in vitro human studies usually use pharmacologically induced epileptiform activity to study the cellular properties of synchrony generating neuronal networks (for review see^2^). All of these interictal discharge models—although they phenomenologically resemble the epileptic interictal spikes—differ from the human disease. Either because epileptic activity is induced by pharmacological or electrical stimulation methods and/or because other mammal species are examined. Furthermore, most human studies describe synchrony generation in the hippocampus and in the surrounding medial temporal areas, whereas neocortical mechanisms leading to pathological synchronisations remain mainly uncovered. In this study, we aimed to examine the role of excitatory and inhibitory neurons (with special focus on the bursting pyramidal cells) in the generation of human neocortical synchronisation mechanisms. Therefore, we investigated the activity of clustered single cells in a human in vitro model of synchronous activity, which generates both physiological population events and epileptiform interictal-like discharges in physiological conditions, without the use of pharmacological agents^3^.

Neuronal behaviour contributing to physiological and pathological synchrony generation has been in the research focus for decades. High numbers of studies emphasized the role of both excitatory and inhibitory cells in the initiation of physiological synchronies. Burst firing CA3 pyramidal cells are known to play a crucial role in generating hippocampal sharp-wave ripple complexes, together with the coordinated firing of inhibitory neurons (for review see^4^). Bursting neocortical layer 5 pyramidal cells initiate up-state, the synchronous activity associated with sleep slow oscillation, involving both excitatory and inhibitory conductances (for review see^5^). Bursting behaviour and paroxysmal depolarisation shift (PDS) of principal neurons seem to play a crucial role in the emergence of pathological synchronies as well (for review see^6^). Especially, intrinsically bursting pyramidal cells were shown to initiate epileptiform activity in acute animal models in the hippocampal CA1^7^, CA3 region^8^ and subiculum^9^, in entorhinal^10^ and somatosensory cortices^11^. In humans, several in vitro^12–16^, and in vivo studies^17–20^ showed the presence of bursts and/or PDS in human neocortical neurons but could not directly relate this cellular behaviour to interictal spike generation. However, an in vivo study demonstrated that neurons participating in interictal spikes have higher bursting rates than non-modulated cells^21^.

More recently, the activity of GABAergic inhibitory networks was also shown to be crucial for the generation of synchronies. Different types of interneurons shape the formation of hippocampal sharp-wave ripple complexes^22,23^, and pharmacologically induced interictal-like discharges^24^ in animals. In humans, inhibitory cells were found to discharge at the beginning of the spontaneously occurring interictal-like activity in the subiculum^25^ and the neocortex^26^, as well as of the epileptiform activity induced by disinhibition in the neocortex^27^. Epileptic seizures associated with low voltage fast activity started with the increased firing of inhibitory cells both in human^28,29^ and experimental epilepsy (for review see^30^), whereas seizures with hypersynchronous spike and waves were related to the enhanced activity of excitatory circuits^31,32^. Although the firing of GABAergic cells can induce depolarizing actions in the epileptic subiculum^25^, this phenomenon affects only a subset of the postsynaptic pyramidal cells, depending on their impaired chloride homeostasis^33^. The lack of inhibition was also shown in human epilepsy both in the peritumoural neocortex^14^ and the hippocampus^34^, but there is evidence for the presence of functional inhibition in both the epileptic neocortex^3,35^ and hippocampus^36^.

Chronically implanted intracortical microelectrodes used for monitoring neuronal activity are only used in epileptic patients (for review see^37^). In non-epileptic subjects such data cannot be obtained. In vitro studies have a considerable advantage in this point: epileptic neocortical tissue can be compared to samples obtained from patients who did not show electrographic or clinical manifestations of epileptic seizures before their brain surgery. Disadvantages of in vitro conditions, however, are evident compared to in vivo recordings, i.e. the input and output connections of the neocortex are cut, and the ionic/molecular composition of the surrounding bath is unquestionably different from the cerebro-spinal fluid. These circumstances might affect the spontaneous firing pattern of neurons, since their synaptic connections are considerably altered. Nevertheless, cell firing and synchronisation mechanisms in epileptic tissue can be compared to non-epileptic cellular and network activity under the same conditions.

Here, we investigated the activity of individual neurons in the human neocortex in an in vitro model spontaneously generating both physiological synchronous population activity (SPA) and experimental interictal-like epileptic discharges (eIEDs). We describe the firing properties of excitatory and inhibitory cells both in epileptic and non-epileptic samples, as well as their participation in the generation of synchronies. We conclude that physiological synchronies are the result of a complex interplay between excitatory and inhibitory microcircuits, while epileptic activity is led by excitatory neurons, with the presumable leading role of bursting pyramidal cells.

## Results

### Patient groups

Based on neurological data, we established four patient groups: (1) patients with pharmacoresistant epilepsy (resistant epilepsy, ResEpi), (2) patients with generalized or focal tonic–clonic seizures who were seizure free with appropriate medication (treatable epilepsy, TreatEpi), (3) patients with one generalized tonic–clonic seizure or with occasional (provoked) seizures, and with no need for medication (no medication, NoMed), and (4) tumour patients without preoperative seizures (no epilepsy, NoEpi). Patients in the latter three groups (TreatEpi + NoMed + NoEpi) were operated in order to resect their tumour. Patients in the first three groups (ResEpi + TreatEpi + NoMed) were considered to be epileptic, whereas patients in the NoEpi group will be referred to as non-epileptic (see Suppl. Table 1 and Refs.^3,27^).

### Spontaneous synchronous activities in the human neocortex

**Figure 1:**
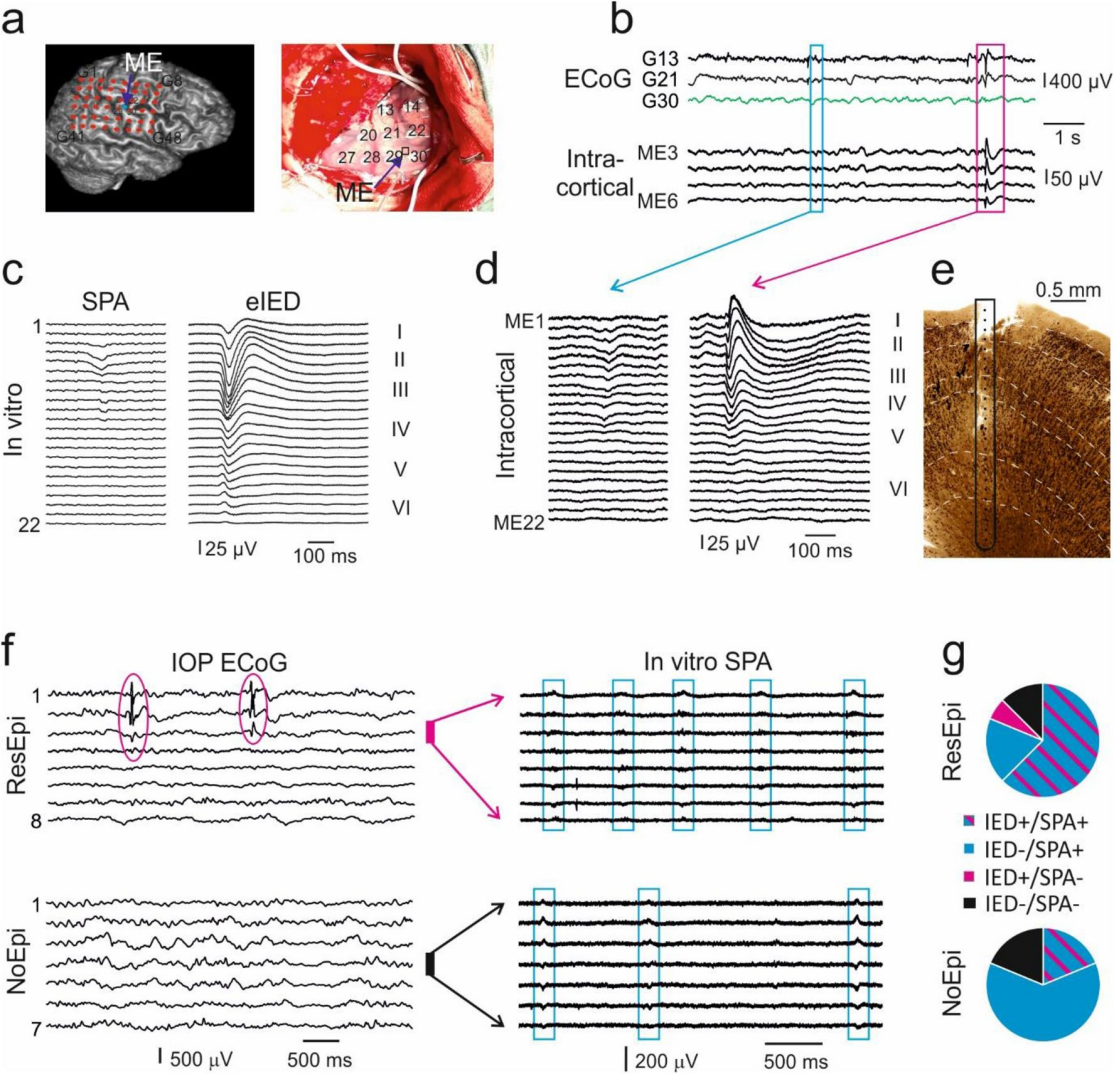
In vitro spontaneous population activity and interictal spikes resemble events recorded in vivo. (**a**) Example showing a multiple channel linear microelectrode (ME) chronically implanted into the neocortex of an epileptic patient below the clinical grid electrode, perpendicularly to the pial surface and covering all layers of the neocortex. (**b**) The clinical grid electrodes shown on (**a**) recorded the electrocorticography (ECoG) simultaneously with the local field potential gradient detected by the ME. (**c**) Two types of spontaneous synchronous activities were recorded in human neocortical slices in a physiological bath. In vitro experimental interictal-like epileptic discharge (eIED) emerged only in epileptic tissue and showed similar waveforms to in vivo recorded interictal spikes ((**b**) magenta box, intracortical magnified on (**d**)). Lower amplitude synchronous population activity (SPA on (**c**)) was generated in slices derived from both epileptic and non-epileptic patients (this example was detected in the tissue sample resected from the patient shown in (**a**, **b**)). Similar synchronous events were identified in intracortical recordings (**b**, light blue box, intracortical magnified on (**d**)). (**f**) In vitro SPA emerged in specimens with and without interictal epileptic discharges (IED, magenta circle) observed on the intraoperative ECoG (IOP ECoG), both in ResEpi and NoEpi tissue. The thick magenta line shows the location of the tissue sample obtained from a ResEpi patient, generating SPA in postoperative slices. The thick black line shows the location of a NoEpi tissue specimen which initiated SPA in vitro, during the postoperative experiment. (**g**) Ratio of tissue samples showing or not showing in vivo intraoperative IEDs (IED+ and IED−, respectively) in comparison with its ability to generate (SPA+) or not generate (SPA−) SPA in postoperative in vitro experiments. Note that the majority of the samples without IED were generating SPA in NoEpi patients (blue).

Human neocortical slices generated two different types of synchronous activities. Similar to interictal epileptic discharges (IED) recorded in vivo with chronically implanted intracortical microelectrodes in ResEpi patients, large and complex synchronous bursts emerged spontaneously in slices derived from epileptic (ResEpi and TreatEpi) patients (Fig. 1, “Supplementary material”). These in vitro interictal-like spikes (experimental interictal-like epileptic discharge, eIED) had similar waveforms as their in vivo counterparts, were characterised by elevated excitability and synchrony, and were considered to be epileptiform events^3,27^. Furthermore, significantly lower amplitude synchronous population activity (SPA) emerged in neocortical slices derived from all four patient groups (see also^3,27^). Similar synchronous events were detected on the chronic intracortical recordings performed in the neocortex of all six examined patients (Fig. 1, “Supplementary material”). These in vivo synchronous events were different from the typical spike-and-wave shaped IEDs and were comparable to in vitro SPAs. They were recorded either in supragranular or in all neocortical layers and—possibly due to their low LFPg amplitude—could not be identified on the ECoG. Note, that SPA and eIED designates the totality of the recurring synchronous events in a given recording. Single population events will be referred to as ‘SPA event’ and ‘eIED event’. The spontaneously recurring in vitro SPA was thought to be a physiological synchronous event^3,27^, as it was generated in tissue derived from NoEpi patients as well. Since brain tumours—mostly those of glial origin—are known to be highly epileptogenic^38^, we cannot exclude the possibility that tumour patients had an overlooked epilepsy. Therefore, we checked the epileptogenicity of the neocortex by performing intraoperative electrocorticography (ECoG) in 41 patients (16 ResEpi, 6 TreatEpi, 4 NoMed and 15 NoEpi patients) on the brain area to be resected, and compared the presence of intraoperative interictal epileptic discharges to the emergence of SPA in vitro. IEDs were detected on the ECoG in 15/16 ResEpi, 4/6 TreatEpi, 1/3 NoMed and 5/15 NoEpi patients. In all patients, IEDs were observed only on a subset of the ECoG electrode contacts. We recorded IED activity on the electrode contact located above the subsequently obtained tissue sample and related it to the presence of SPA detected in vitro (Suppl. Table 2, Suppl. Fig. 2). In vitro SPA was generated in the large majority of the samples (Fig. 1f,g). In total, 13/16 ResEpi and 13/16 NoEpi specimens initiated SPA in vitro. 10/11 ResEpi and 3/3 NoEpi samples showing in vivo IED generated SPA in vitro, and 3/5 ResEpi and 10/13 NoEpi samples without the signs of IED also generated SPA in vitro (for details see “Supplementary material”). Thus, SPA is clearly generated by the neocortex of both epileptic patients with IED and non-epileptic patients without IED activity.

**Figure 2:**
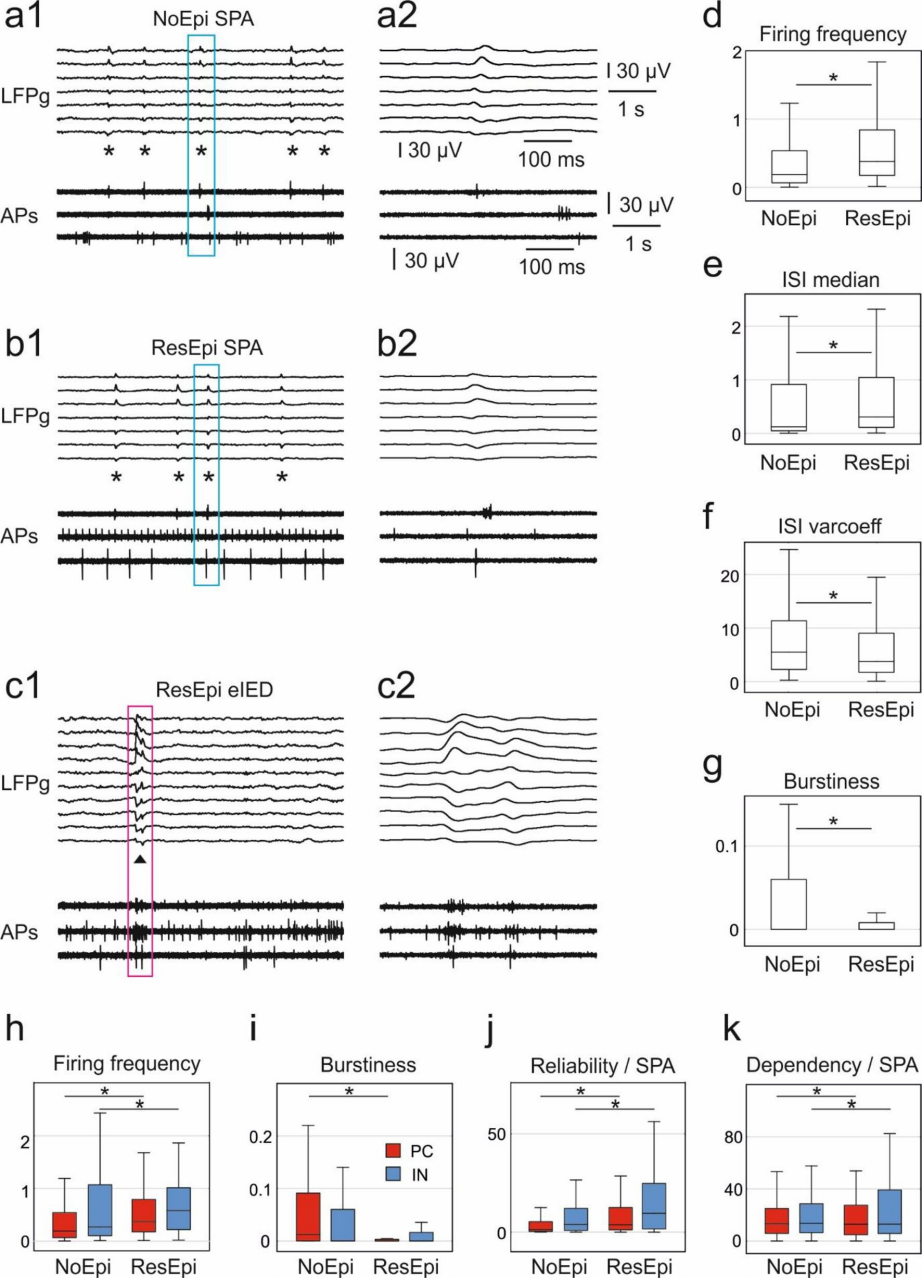
Synchronies and cellular firing in the human neocortex, in vitro. Spontaneous population activity (SPA, asterisks) was recorded in human neocortical slices derived from NoEpi (**a**) and ResEpi (**b**) patients. Additionally, larger and more complex interictal-like discharges (eIEDs, triangles on (**c**)) emerged from epileptic tissue, in vitro. Upper traces show the band-pass filtered (1–30 Hz) local field potential gradient (LFPg), lower traces confirm the presence of action potentials of single cells (APs, high pass filtered at 500 Hz). The events in the boxes are magnified on the right panels ((**a2**–**c2**)). Both the average firing frequency (**d**) and median inter-spike-interval (ISI) of neurons (**e**) in ResEpi were higher than in NoEpi tissue (p < 10^–09^ and p < 10^–05^, respectively). The variation coefficient of the ISIs (**f**) and the burstiness of cells (**g**) were lower in ResEpi than in NoEpi slices (p < 0.001 and p < 10^–4^, respectively). The firing frequency (**h**) of both PCs (red) and INs (blue) was higher (p < 10^–4^ for both) in ResEpi compared to NoEpi slices. The burstiness (**i**) of PCs (but not that of INs) was significantly lower (p < 10^–4^) in ResEpi than in NoEpi tissue. The reliability of the neuronal firing during SPA ((**j**), % of SPA events with cell firing) was elevated in ResEpi tissue compared to NoEpi (p < 10^–8^ for PCs, p < 10^–4^ for INs), such as the neurons’ dependencies on SPAs ((**k**), % of APs during SPA events, p < 0.05 for both cell types).

In epileptic patients, the occurrence rate of SPA was linked to the aetiology: it was significantly lower in slices from patients with tumour than from patients with dysgenesis or hippocampal sclerosis or other pathology^3^. We explicitly chose NoEpi specimens as well as ResEpi cases without tumour for the neuronal firing dynamics analysis (for patient data see Suppl. Table 1), to assess changes linked to epilepsy. Furthermore, we excluded TreatEpi and NoMed patients from these analyses, since they represent very heterogeneous groups regarding the duration of epilepsy, seizure occurrence and antiepileptic medication. The recurrence frequency of ResEpi SPAs was similar to that of NoEpi SPAs but was higher than that of ResEpi eIEDs (ResEpi SPAs and eIEDs were significantly different, p < 10^–4^; common language effect size, ES = 0.98). The local field potential gradient (LFPg) amplitude of the events was significantly higher for ResEpi SPA than for NoEpi SPA (p < 0.01, ES = 0.69), and even more elevated for eIEDs (significantly different from ResEpi SPA, p < 0.001, ES = 0.95, Fig. 2a–c). The multiple unit activity (MUA) was similar for NoEpi and ResEpi SPA and was higher for eIED (significantly different from ResEpi SPA, p < 0.01, ES = 0.85; for amplitudes/values see “Supplementary material”).

### Cellular characteristics—clustering data

We explored cellular characteristics and single neuron activity during synchronous activity. We clustered and examined 406 neurons in 29 recordings derived from NoEpi patients, and 351 cells in 37 recordings from ResEpi tissue (Suppl. Table 4). In NoEpi tissue we separated 132 principal cells (PC, 32.5% of all cells), 104 interneurons (IN, 25.6%), and could not classify 170 cells (UC = unclassified cells, 41.9%). In ResEpi tissue we separated 119 PCs (33.9%), 102 INs (29.1%) and 130 UCs (37.0%, see “Methods”).

The average firing frequency of all neurons—independently of the SPA/eIED at the recording site—was significantly higher in ResEpi slices (0.37 [0.16–0.83] Hz) than in NoEpi slices (0.17 [0.06–0.53] Hz, p < 10^–9^, ES = 0.63). Both PCs and INs had a significantly higher firing rate in ResEpi than in NoEpi tissue (Fig. 2d,h, Suppl. Table 4). As in the human neocortex in vivo^39^, we saw a tendency for higher firing frequencies in INs compared to PCs, both in ResEpi and NoEpi samples. However, INs had a significantly larger median inter-spike-interval (ISI, note that spike means action potential) on average than PCs, both in NoEpi and in ResEpi tissue. The median ISI was also larger in ResEpi cells (0.30 [0.11–1.09] s) than in NoEpi cells (0.13 [0.05–0.99] s, p < 10^–5^, ES = 0.59), as well as the mean firing frequency (see above). The apparent contradiction of the larger ISI with the larger average firing frequency can be explained by more irregular firing in NoEpi tissue (see “Supplementary material”), which is also indicated by a smaller ISI variation coefficient in the ResEpi group (3.71 [1.74–8.77]) compared to the NoEpi group (5.47 [2.28–11.34], p < 0.001, ES = 0.57, Fig. 2).

Bursting behaviour of neurons has been linked to epileptogenesis both in the human^12^ and rodent^11^ neocortex. We differentiated between synaptically driven bursting behaviour, i.e. the ability of neurons generating clusters of APs in response to synaptic input, and intrinsic bursting, i.e. a neuron’s tendency to fire bursts solely as a manifestation of its intrinsic membrane properties and independent of its synaptic input (for review see^40^). Although synaptically driven and intrinsic bursting were originally determined in intracellular recordings together with extracellular electric stimulation and intracellular current injection^41^, the properties of the spontaneous firing pattern of individual neurons in extracellular records can give an estimate of synaptically driven and intrinsically bursting behaviour of the neurons. In our study, synaptically driven bursting was assessed by quantifying the burstiness index of the neurons, meaning the percentage of APs within bursts during the whole recording. Background periods represent lower, whereas SPA/eIED events correspond to higher synaptic input^3,25,33^ (see “Methods”). Intrinsic bursting was estimated from the autocorrelogram, by defining whether a principal cell shows a clear intrinsically bursting (IB), regular spiking (RS) or unclear pattern, i.e., the tendency of the neuron to fire in bursts vs. in repetitive single action potentials^42,43^ (see “Methods”). Interestingly, we found that neurons in the ResEpi neocortex had a significantly lower synaptically driven burstiness (0.00 [0.00–0.96]) than cells in slices derived from NoEpi patients (0.00 [0.00–6.00], p < 10^–6^, ES = 0.59, Fig. 2g,i, Suppl. Table 4). This was valid for PCs (p < 10^–4^, ES = 0.65), but not for INs (p > 0.5, ES = 0.54). When analysing the different PC types, we found that the burstiness of RS-PCs and unclear firing PCs was significantly lower in NoEpi than in ResEpi tissue, but not that of IB-PCs (Supplementary Table 7). Furthermore, as expected^44^, PCs fired more bursts than INs, although this effect was only observed in NoEpi (p < 0.001, ES = 0.65), but not in ResEpi samples (p > 0.4, ES = 0.55). The ratio of IB-PCs was 10.1% of all PCs in ResEpi and 15.2% in NoEpi tissue, similar to the rodent neocortex^45,46^. However, no significant differences (p > 0.2) were found in the numbers of PCs with different firing patterns between NoEpi and ResEpi samples.

### Firing behaviour during synchronies

**Figure 3:**
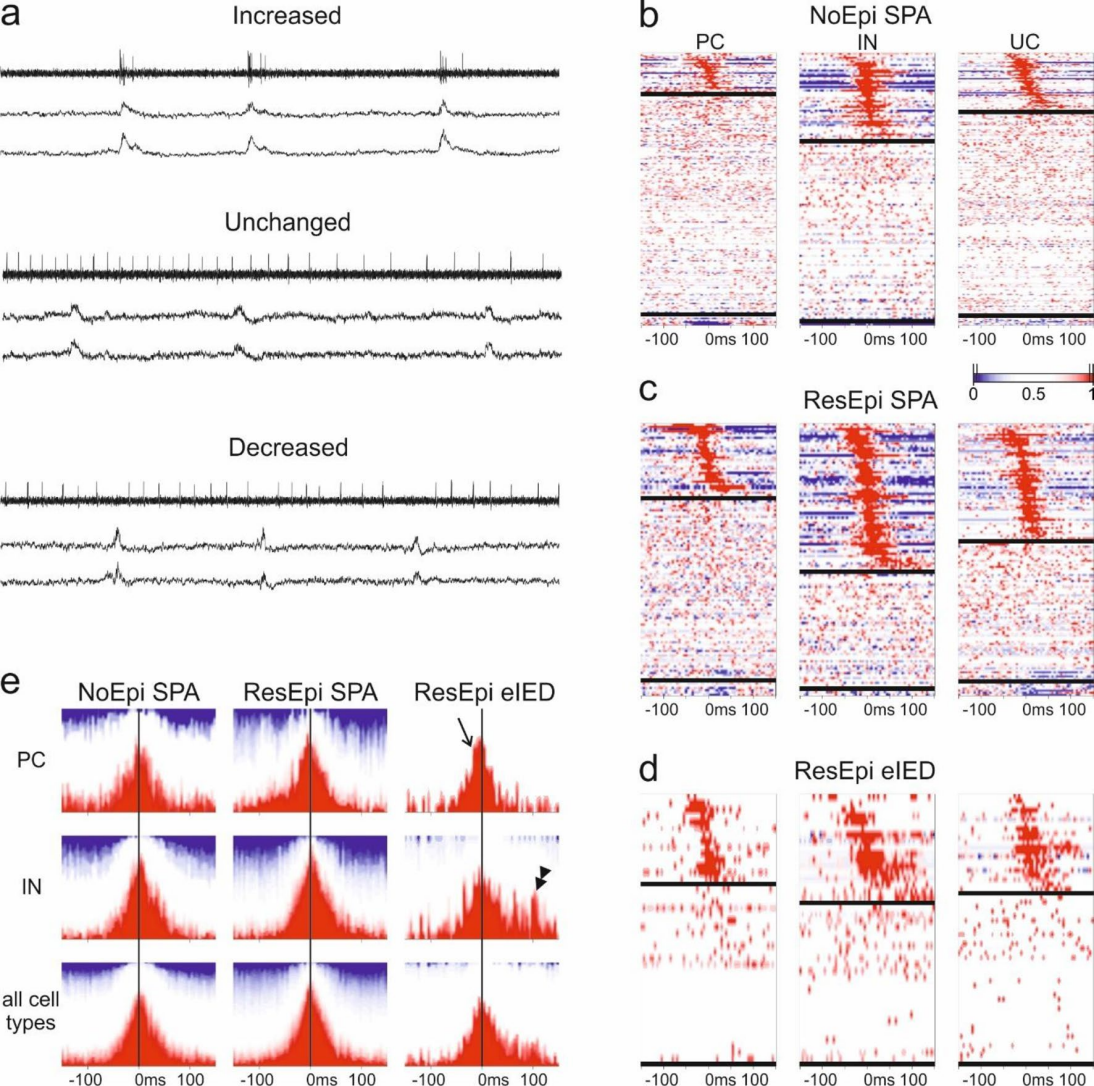
Firing rate change during SPA and eIED. (**a**) Examples for neurons with increased, unchanged and decreased firing rate during SPA. Upper traces show the activity of the neuron, lower two traces show the SPA (filters as in Fig. 2). Firing change quantiles of all neurons around the LFPg peak (time 0) of NoEpi SPA (**b**), ResEpi SPA (**c**) and ResEpi eIED (**d**). The three columns reflect principal cells (PC), interneurons (IN) and unclassified cells (UC), respectively. Each row illustrates one cell-SPA/eIED relation. Red shows increase, blue marks decrease in firing. Vertical black lines on the scale bar depict significant intervals. Horizontal black lines separate neurons with increased (upper part), unchanged (middle part) and decreased (lower part) firing. Higher proportions of PCs and INs elevate their firing rate during ResEpi than NoEpi SPA. Increased cells are sorted based on the timing of their maximal firing relative to the SPA/eIED. Note, that all cell types can increase their discharge rate at the beginning and at the end of the events. (**e**) Summary of firing change quantiles of all increased cells around the LFPg peak of NoEpi SPA (left), ResEpi SPA (middle) and ResEpi eIED (right). The peak firing of PCs during eIEDs precedes the LFPg maximum of the events (open arrow). INs (second row) show a symmetric build-up and decline during SPAs (both NoEpi and ResEpi), while displaying elevated firing long after the peak of eIEDs (double arrowhead). Considering the summarized activity of all increased cells of all cell types (third row), SPAs are symmetric, eIEDs are asymmetric events.

The firing behaviour of the clustered neurons was examined during SPAs and eIEDs (Fig. 3, Suppl. Table 5). We made 603 and 360 cell/SPA comparisons in NoEpi and ResEpi tissue, respectively, and 141 cell/eIED comparisons in ResEpi tissue.

For determining the significance of a potential cell firing rate increase/decrease during SPAs/eIEDs, we computed the firing change quantile rather than the firing change itself, in order to avoid false outcomes resulting from low numbers of cell APs (see “Methods” and “Supplementary material”). In the ResEpi group, 41.9% and 36.9% of cells showed an increased firing rate during SPAs and eIEDs, respectively, whereas it was only 21.7% during NoEpi SPAs (Fig. 3). The number of cells with decreased firing rate was around 2–4% during SPAs, comparable between ResEpi and NoEpi tissue. No cells were found to significantly decrease their firing rate during eIEDs, but this might be related to the low number of eIED events and the resulting low statistical power. The proportions of increased/unchanged/decreased cells during SPA between NoEpi and ResEpi were significantly different (p < 10^–10^). The post hoc test indicated a higher number of cells with increased firing rate in ResEpi compared to the NoEpi group. The firing change quantile of all cells was significantly lower in NoEpi SPA (0.64 [0.34–0.95]) than in ResEpi SPA (0.92 [0.53–1.00], p < 10^–10^, ES = 0.63), in ResEpi eIED it was 0.81 [0.44–1.00].

Next, we examined how excitatory PCs and inhibitory INs discharged during human neocortical synchronies. Inhibitory cells tended to participate in synchronous events at higher proportions than principal cells: during NoEpi SPA 14.7% of the PCs but 32.6% of the INs showed a significantly increased firing rate. In ResEpi, this pattern was also found. Moreover, compared to NoEpi, more cells showed a significantly increased firing rate in ResEpi, both during SPA (27.5% of the PCs and 55.5% of the INs), and during eIED (33.3% of the PCs and 40.5% of the INs; Fig. 3b–d). Also, the firing change quantile was significantly higher for all INs than for all PCs during ResEpi SPA. Furthermore, for both PCs and INs the firing change quantiles were higher in ResEpi than in NoEpi SPA (Suppl. Table 5).

We verified how neuronal firing is attributed to synchronies, by determining the reliability (% of SPA/eIED events with cell discharge) and the dependency (% of APs happening during SPAs/eIEDs) of neurons during SPAs and eIEDs. We found that the reliability of neuronal firing was higher in ResEpi SPA (4.42 [1.82–15.79]) than in NoEpi SPA (1.33 [0.25–6.25], p < 10^–19^, ES = 0.68, Fig. 2). INs fired more reliably than PCs during both NoEpi and ResEpi SPA, and all cell types showed higher reliability values during ResEpi than NoEpi SPA. Neuronal firing reliabilities during eIED were comparable to those found in ResEpi SPA. The dependency of cellular discharge was also higher in ResEpi SPA (18.81 [9.24–34.15]) than in NoEpi SPA (13.43 [5.49–25.0], p < 10^–08^, ES = 0.61). In accordance with the very low number of eIED events/recording, the dependency of cellular firing was significantly lower during eIEDs (1.79 [0.0–6.27], p < 10^–30^, ES = 0.83) than during ResEpi SPAs. In summary, neurons in ResEpi tissue are more closely linked to SPA than in NoEpi tissue (higher reliability and higher dependency), and INs tend to participate in synchronies with a higher reliability than PCs.

### Initiation of SPA/eIED

Data obtained in epileptic patients indicated that neuronal interactions and network mechanisms drive the generation of interictal spikes^21^. In human neocortical slices, inhibitory interneurons were suggested to initiate both spontaneous^26^ and disinhibition-induced^27^ interictal-like activity, while pyramidal cells followed. We aimed to look at cellular interactions during SPAs and spontaneously occurring eIEDs by using several different approaches. First, we determined the time point of the maximal firing rate relative to the SPA/eIED peak for every cell. This value was very variable with a median around 0 ms for all types of neurons in both ResEpi and NoEpi tissue, during both SPAs and eIEDs (Suppl. Table 5). Second, we combined the firing change quantiles of all cells for each 5 ms time bin around synchronies and examined PC and IN firing at the population level (Fig. 3b–e). We could observe certain differences between NoEpi SPAs, ResEpi SPAs and eIEDs. For both NoEpi and ResEpi, the firing of all cells showed symmetric build-up and decline periods during SPAs, whereas it was asymmetric during eIEDs. Especially, INs displayed a symmetrical firing increase and decrease during NoEpi and ResEpi SPA, but showed an elevated discharge during the decline period of eIEDs.

**Figure 4:**
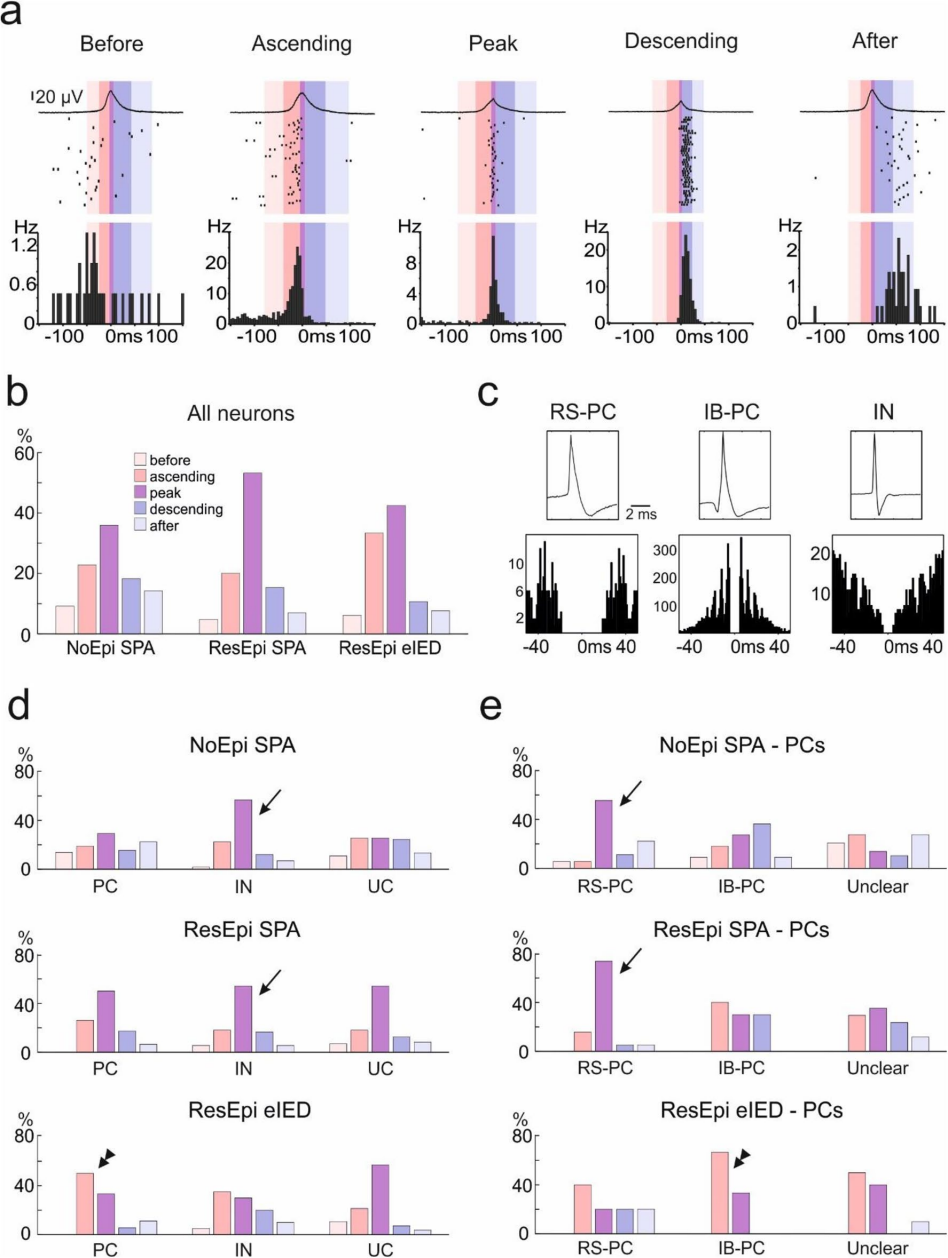
Relative firing change during SPAs and eIEDs. The firing change of neurons was examined during the different phases of SPA/eIED. (**a**) Examples of cells with increased firing during before, ascending, peak, descending and after phases of synchronous events. The upper trace shows the average LFPg of the respective SPA, the raster plot displays randomly chosen sweeps of the cell, and the peri event time histogram (bottom) averages the firing of the cell. Coloured areas mark the different phases of the event (for colour coding see inset in (**b**)). (**b**) Percentages of cells with increased firing rates during the different phases of SPAs/eIEDs. Note the high proportion of ‘ascending’ cells during eIEDs. (**c**) Regular spiking (RS-PC) and intrinsically bursting (IB-PC) PCs, as well as INs were identified based on the waveform of their APs (upper panel) and their autocorrelogram (lower panel). (**d, e**) High numbers of interneurons (arrows on (**d**)) and regular spiking PCs (arrows on (**e**)) increased their firing rate during the peak phase of SPAs (both NoEpi and ResEpi, upper and middle panels, respectively), while PCs (double arrowhead on (**d**)), especially intrinsically bursting PCs (double arrowhead on (**e**)) preferred to elevate their firing rate during the ascending phase of eIEDs (lower panel).

Next, we divided SPAs/eIEDs into five phases (before, ascending, peak, descending, after; see “Supplementary material”) and examined neuronal firing within these time intervals. We found 198/603 cells (32.8%) during NoEpi SPA, 190/360 cells (52.8%) during ResEpi SPA and 66/141 cells (46.8%) during ResEpi eIEDs with increased firing rates during one of these periods (Fig. 4, Suppl. Table 6). Most neurons fired maximally during the ascending, peak or descending phases of the events, whereas only few cells showed increased firing before or after the SPAs/eIEDs. Both PCs and INs were found to have elevated firing rates during the ascending, peak and descending phases of both types of synchronous events, both in ResEpi and in NoEpi tissue. The distribution of cells with increased firing in the five different phases was different between ResEpi SPA and NoEpi SPA, with higher proportions of cells showing maximal firing during the peak in ResEpi SPA than in NoEpi SPA (Fig. 4). Compared to SPAs, during eIEDs neurons tended to discharge more during the ascending phase.

Bursting behaviour of neurons has been related to interictal^47^ and preictal^48^ spike generation. We thus checked whether the synaptically driven bursting behaviour or the intrinsic bursting of the cells is related to synchrony generation. Neurons responding with increased firing to SPAs had a synaptically driven burstiness index comparable to that of decreased and unchanged cells (both in ResEpi and NoEpi). In contrast, similar to in vivo conditions^21^, neurons with increased firing rates during eIEDs showed a significantly higher burstiness than non-increased cells (p < 0.05).

To get information about how intrinsic bursting behaviour is associated with synchrony initiation, we analysed how IB-PCs, RS-PCs and INs participated in the generation of synchronies (Fig. 4). During NoEpi and ResEpi SPA we found high participation of RS-PCs and INs during the peak of the event. During ResEpi eIEDs, cells behaved differently. INs and RS-PCs were not closely linked to the peak of eIEDs as during SPAs. The large majority of the PCs discharged during the ascending phase (9/18 of participating PCs) or the peak (6/18 of increased PCs) of eIEDs. Two out of the three increased firing IB-PCs were associated to the ascending phase, and one to the peak of the eIED events. All participating IB-PCs were discharging in bursts during 42 ± 7% of the eIED events, while they fired only single APs between the events. Furthermore, 70% of the participating unclear firing PCs were also producing bursts during 9 ± 9% of the eIED events while firing only single APs between the events. All of these neurons were linked to either the ascending phase (50%) or the peak (50%) of the eIED events. RS-PCs never fired bursts during eIED.

**Figure 5:**
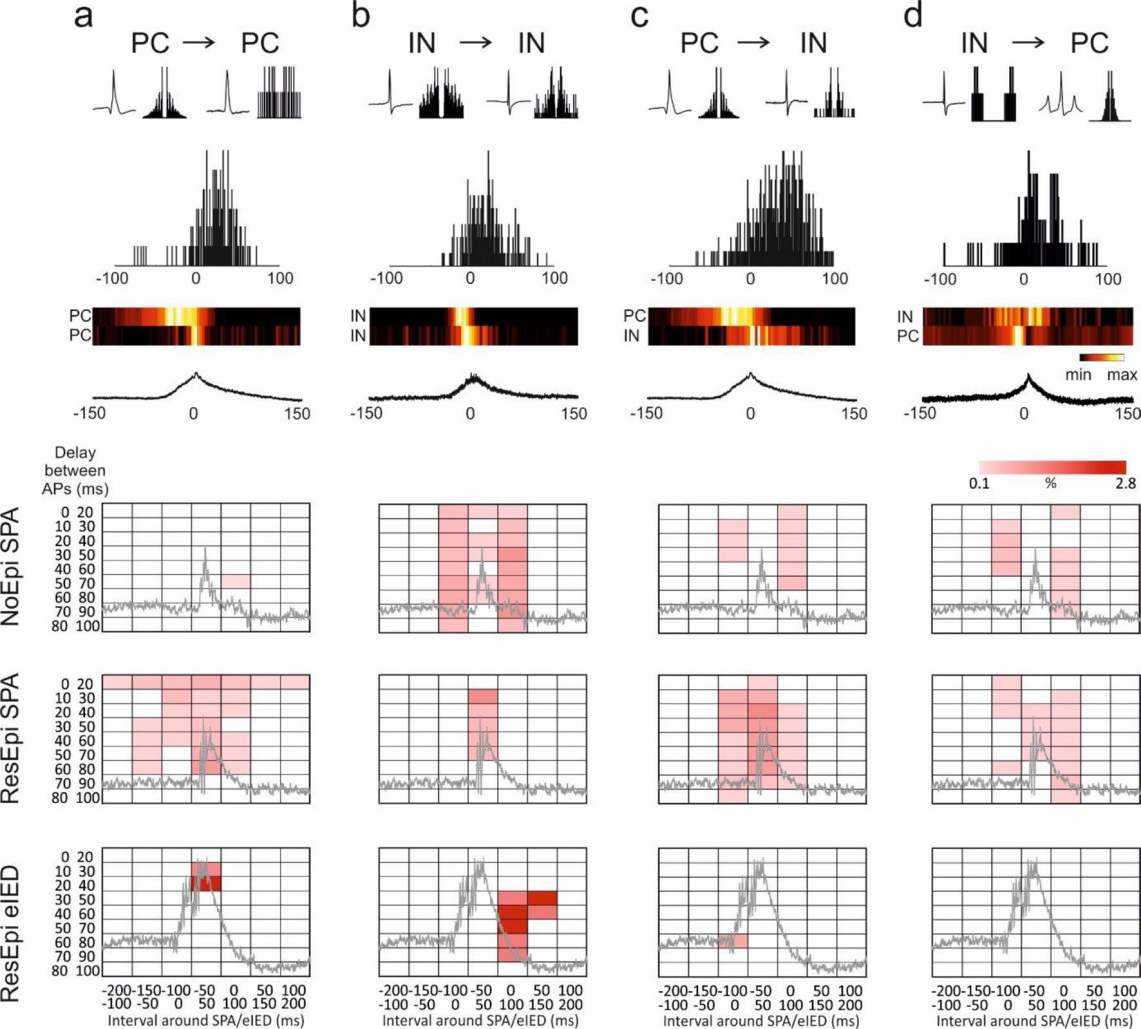
Cellular interactions during SPA and eIED. We examined how neuronal firing affects discharge in other cells. Examples for PC to PC (**a**), IN to IN (**b**), IN to PC (**c**) and PC to IN (**d**) firing sequences. (**a–d**) AP waveform and the auto-correlogram of the two respective cells are shown in the first row, the asymmetric cross-correlogram in the second row. The heat maps show the firing of the cells relative to the respective SPA (lower trace). Hot colours depict increased firing frequency (min: minimal, max: maximal firing rate). Note that the bursting PC in (**a**) is the same as the PC on (**c**) driving both a PC and an IN. The lower panels show the percentages of significant cellular interactions, checked at different, 100 ms long time intervals around the LFPg peak of the SPA/eIED events (between − 200 and + 200 ms, x-axes), as well as at different delays between the cells (20 ms long delay intervals, between 0 and 100 ms, y-axes). Only one PC-PC interaction was found during NoEpi SPA (first panel), while numerous PC-PC interactions characterised ResEpi SPA (second panel). The timing of all types of neuronal interactions was mostly symmetric around the LFPg peak of NoEpi SPA (first row panels) and ResEpi SPA (second row panels), while it was asymmetric around the peak of eIEDs (bottom row panels). Around ResEpi eIED (bottom row panels) PC-PC interactions were shorter in delay (up to 40 ms) and concentrated at the peak, while IN-IN interactions appeared after the peak of the eIEDs (0–150 ms) with a higher delay (up to 90 ms). During NoEpi SPA (first row panels) PC-IN and IN-PC interactions appeared equally around the peak. During ResEpi SPA, higher numbers of PC-IN than IN-PC interactions were observed. Light pink to dark red rectangles depict increased percentages of significant cell–cell interactions. For exact numbers see Suppl. Table 8.

As another approach, we examined the interactions of single cells, as well as their firing sequence during synchronies, by computing cell–cell cross-correlograms (see “Methods”). Note, that only asymmetric cell–cell interactions are detected by this method, the reciprocal connection between two cells in the range of the comparison will not be uncovered. Cellular interactions were only examined in the temporal vicinity of the SPAs/eIEDs. During NoEpi SPAs, significant IN–IN, IN-PC and PC-IN interactions were distributed symmetrically within ± 100 ms around the LFPg peak of the SPA (Fig. 5, Supplementary Table 9). Interestingly, we revealed only one significant PC-PC interaction. In ResEpi SPA, considerably higher numbers of PC-PC and PC-IN, but fewer IN-IN interactions were detected. During eIEDs, PC-PC interactions were concentrated to the peak (within ± 50 ms), while IN-IN interactions were observed after the peak (0–150 ms) of the events. Only one significant PC-IN was found during eIEDs, in the range of 0–100 ms after the peak of the eIED.

## Discussion

### Physiological and pathological synchronies

Human neocortical slices generated two different types of synchronous events: eIED and SPA (see also^3^). eIED appeared only in specimens derived from epileptic patients, in the granular and infragranular layers of the neocortex. They were hypersynchronous bursts characterized by an elevated excitability, and resembled in vivo^49^ and pharmacologically induced in vitro^27,50^ interictal spikes. SPAs emerged in both epileptic and non-epileptic tissue, mainly in the supragranular layers, and were synchronous events with significantly lower levels of synchrony and excitability^3^. Both types of synchronous events could be identified in chronic intracortical recordings with comparable waveform, amplitude and location. Intraoperative ECoG recordings confirmed the hypothesis that SPA is not related to epilepsy, since it was generated in brain samples of patients without preoperative seizures and with no interictal spiking activity on their ECoG.

Several research groups detected spontaneously occurring population bursts in neocortical slices derived from epileptic patients^26,51–54^, and related them to the epileptogenicity of the resected tissue. However, based on the events’ network and cellular properties (location within the neocortex, recurrence frequency, waveform of the activity, increased high frequency oscillations and cellular firing, participation of glutamatergic and GABAergic signalling, and intracellular correlates), these synchronous burst activities share similarities with our SPA, and differ from both spontaneous^3^ and evoked^27,50^ interictal-like discharges generated in human epileptic neocortical slices.

SPA seems to be a human-specific phenomenon, since no similar synchrony could be detected in neocortical slices of any other species. It emerged mainly in the supragranular layers, such as the large complex synaptic event sequences^55^, K-complexes^56^, theta, gamma^57^ and sleep slow oscillation^58^. Recently, human-specific connectivity rules^59^ and a neuron type^60^ were described in the supragranular layers of the human neocortex, which underline the differences between humans and non-human mammals and might account for the accentuated role of this neocortical lamina in synchrony generation.

### Cellular behaviour during physiological synchronous population activity

**Figure 6:**
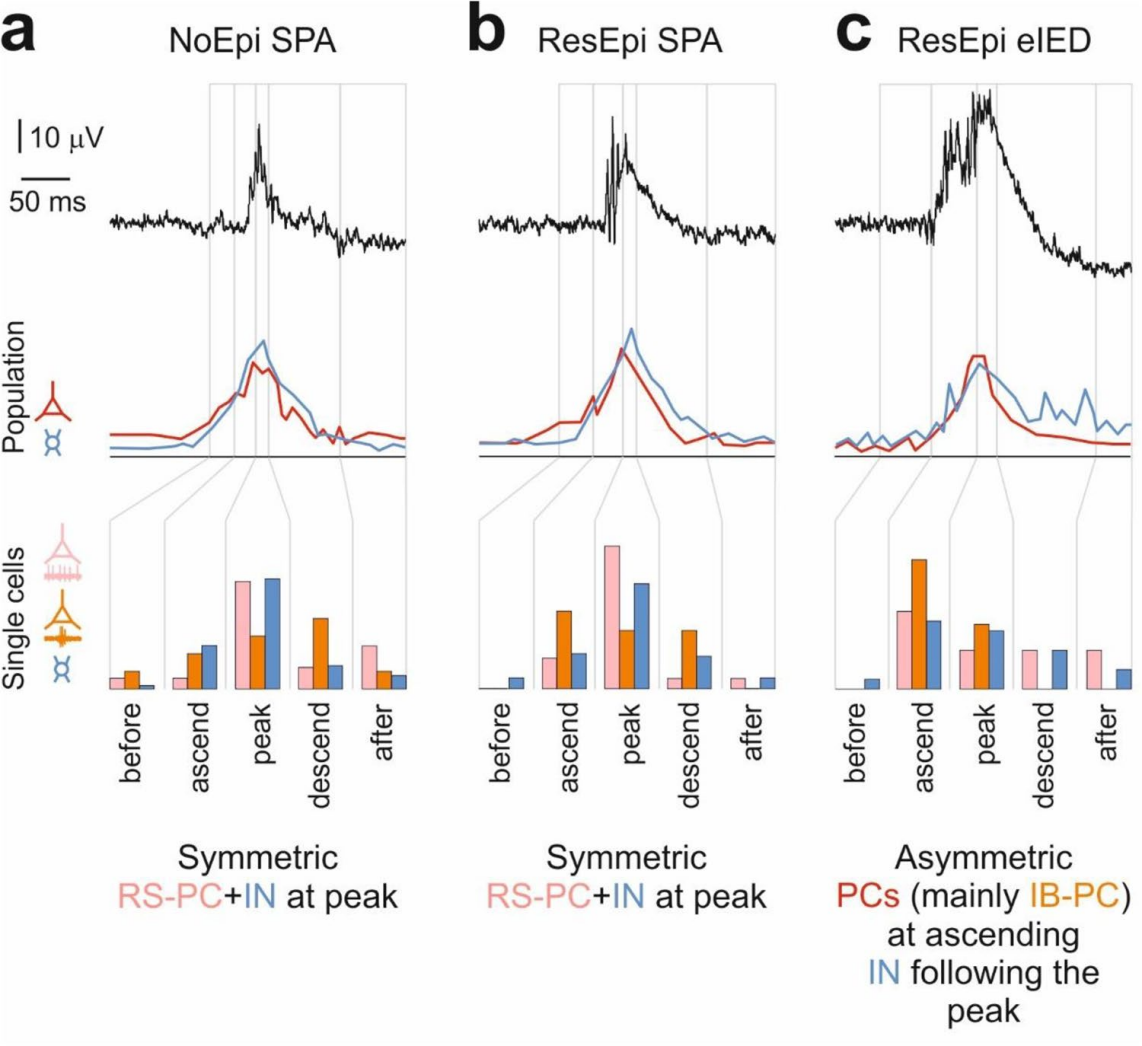
Cellular mechanisms of physiological SPAs and pathological eIEDs. This figure summarises the excitatory (reds) and inhibitory (blue) cellular processes during physiological (**a**, **b**) and pathological (**c**) synchronies. Upper traces show a broad-band recorded example for NoEpi SPA (**a**), ResEpi SPA (**b**) and eIED (**c**). Note that the after phases are cropped in the figure. Grey lines demarcate the five phases of synchronous events. Line graphs show the summed firing of the PC (red) and IN (blue) populations. Bar graphs show the elevated firing of regular spiking (RS, light pink) and intrinsically bursting (IB, orange) PCs as well as INs (blue) during the five phases of synchronous events. During ResEpi eIEDs INs show a prolonged increase in firing after the LFPg peak. The activity of RS-PCs and INs is highly connected to the peak phase of SPAs (both NoEpi and ResEpi). IB-PCs seem to fire mostly during the descending phases of NoEpi SPAs, ubiquitously during ResEpi SPAs and mostly during the ascending phase of ResEpi eIEDs. A largely symmetric build up and decline in firing was observed during SPAs, while the asymmetry of IB-PC and IN activity resulted in an asymmetric firing during eIEDs.

Excitatory and inhibitory neurons equally participated in the generation of physiological SPA (Fig. 3). We observed both PCs and INs to increase their firing rate during all phases of the SPA. When comparing PC-IN interactions during the SPA, we observed that both cell types can start firing while the other follows. Our firing pattern analysis suggested a gradual build-up and decline of neuronal discharge during SPA, involving all cell types (Fig. 6). Similar mechanisms operate during physiological synchronies in animals. The firing of excitatory pyramidal cells leads the synchronous neuronal activity of slow oscillations during sleep^61^, hippocampal sharp wave ripples^22^ and subicular focal synchronous events^9^, which is followed by a gradual build-up of neuronal activity composed of a complex interplay between excitatory and inhibitory neurons^4,5,9,22^. Human data about physiological synchronisation mechanisms are sparse and contradictory, emphasizing the importance of either excitatory^53,55^ or inhibitory^26^ signalling in initiating SPA-like spontaneous synchronous activity. Our results recall the scenario of the complex synaptic event sequences described in the non-epileptic human neocortex, which are initiated by the firing of single PCs, and recruit both glutamatergic and GABAergic neurons to participate in a synchronous episode^55,62^. The symmetrical and equalized discharge of both excitatory and inhibitory microcircuits during SPA agrees with the dynamic balance of excitation and inhibition found in the human neocortex during non-epileptic brain states, such as the sleep/wake cycle^29^.

On the other hand, we observed certain differences in the cellular firing during SPA, when comparing slices derived from epileptic and non-epileptic patients. Compared to NoEpi samples, ResEpi tissue displayed a higher LFPg amplitude and comprised a significantly larger pool of PCs and INs participating in the generation of SPAs. Moreover, the participating ResEpi cells showed a higher reliability during—and dependency on—SPAs than cells in NoEpi samples. These results indicate that cells in the epileptic neocortex are more involved in the population activity, and are more closely associated to synchrony than cells in non-epileptic tissue. Moreover, higher ratios of PC-PC interactions and PC-IN sequences were detected in ResEpi compared to NoEpi SPA. These results suggest that the finely tuned balance in the activation of excitatory and inhibitory microcircuits has been shifted towards an enhanced excitability and synchrony in epilepsy.

### Cellular behaviour during interictal-like discharges

eIEDs emerging spontaneously in human epileptic neocortical slices are highly synchronous population events characterized by an elevated level of excitability^3^. Contrary to SPAs, an asymmetric overall neuronal firing characterized the eIEDs (Fig. 6). PCs tended to discharge more at the rising phase, while INs showed wider firing patterns extending until after the peak of eIEDs. This asymmetry was also reflected in the cellular interactions, with high numbers of PC-PC and IN-IN, but almost no PC-IN interactions. Both excitatory and inhibitory circuits were involved, with a clear leading role of (bursting) PCs. These results support the hypothesis that eIEDs are complex and dynamic network phenomena, similar to the in vivo situation^21^. The idea of balanced excitatory and inhibitory neuronal dynamics underlying physiological population events^63^, becoming perturbed^29^ and temporally asymmetric during epileptic synchronisations appears in previous articles investigating the human^48^ and the rodent hippocampus^24^. However, such a temporal asymmetry in the excitatory and inhibitory firing during interictal spikes was not described yet.

The role of excitatory and inhibitory circuits in the initiation of epileptic activities depends on the examined synchrony type, model and species. Seizures with hypersynchronous spike onsets were driven by excitatory processes^31,32^, whereas low voltage fast activity seizures were characterized by the dominance of inhibitory cell firing^28,29,64^. Depolarizing GABAergic signalling initiated the events in a pharmacologically induced acute model of IED^65^, while the firing of glutamatergic neurons was shown in others^8,24,66^. Furthermore, disinhibition-induced eIED was initiated by bursting PCs in the rodent neocortex^11^, whereas an intense interneuronal discharge was observed in the same model in the human neocortex^27^. Our results draw attention to the differences between experimental models and the human disease. Although the morphology of the events might be similar, the mechanisms of spontaneously occurring eIED clearly differ from pharmacologically induced epileptiform episodes.

### Bursting behavior and epilepsy

Several decades ago, bursting behaviour of neurons was linked to epilepsy both in animals^11^ and humans^12^, in vitro. Studies using microelectrode recordings in epileptic patients, however, provided contradictory results: both increased^20,44^ and decreased^67^ burstiness has been found in the seizure onset zone of the medial temporal lobe in epileptic patients, compared to the contralateral side, and burst firing could not be directly related to IED generation. Pharmacologically induced preictal discharges in human subicular slices were linked to excitatory signalling which activated burst firing in PCs^48^. To examine the role of bursting cells, we differentiated between synaptically driven bursting (reflected in the burstiness index) and intrinsic bursting (bursting behaviour due to the membrane properties of the cell, estimated from the autocorrelogram)^41^. We found that the burstiness index of neocortical cells (and mostly that of PCs, especially the RS-PCs) in ResEpi tissue was significantly lower than in NoEpi tissue. This might be the result of an altered overall synaptic input^3,68^, synaptic integration^69^ and/or dendritic morphology^70^ of the PCs in epileptic tissue^3^. On the other hand, neurons with increased firing during eIED (but not during SPA) had higher burstiness values than non-participating cells. Furthermore, the extra synaptic input provided by the IED events could elicit burst firing in the majority of unclear firing PCs. These results point to a decreased overall burstiness in the epileptic tissue together with an increased bursting behaviour specifically linked to eIED. When looking at the intrinsic bursting feature of pyramidal cells, the ratio of IB-PCs was similar in both patient groups. Contrary to the RS-PCs, the burstiness index of IB-PCs was not significantly decreased in ResEpi compared to NoEpi tissue. The discharge of the few detected IB-PCs was markedly connected to the ascending and peak phases of eIED, taking the shape of bursts during most events. This indicates that intrinsic bursting may contribute to interictal activity initiation. Similar to sleep slow oscillation, where a small subset of bursting pyramidal cells acts as “network driver” neurons and initiates up-states^71^, our results suggest that IB-PCs might initiate eIEDs and may provide the extra excitatory synaptic input enhancing the synaptically driven bursting observed during eIEDs.

The differences in the generation mechanisms between SPA and eIED possibly result from a combination of cellular and network characteristics of the human neocortex, including layer specificity^55,60^, the presence of infragranular bursting PCs^41^, as well as from molecular, cellular and connectivity changes related to epilepsy (for reviews see^72,73^).

## Conclusions

Our experiments indicate that physiological synchronous events are characterized by a symmetric and finely tuned balance of excitation and inhibition, which is shifted towards an enhanced excitability in epileptic tissue. In contrast, interictal-like discharges are asymmetric complex network events, mainly initiated by pyramidal cells (Fig. 6). Although the overall synaptically driven burstiness is lower in epileptic tissue, the bursting behaviour of the neurons—composed of both intrinsic and synaptically driven bursting—is clearly linked to interictal epileptiform discharges, suggesting that bursting pyramidal cells might have an important role in initiating interictal spikes in the human neocortex.

## Materials and methods

### Patients

All patients were undergoing brain surgery in the National Institute of Clinical Neuroscience (Budapest, Hungary). They gave informed and written consent, and the study was approved by the Regional and Institutional Committee of Science and Research Ethics of the Scientific Council of Health (ETT TUKEB 20680-4/2012/EKU) and performed in accordance with the Declaration of Helsinki. The patients included in this study are partly overlapping with the patient sets described in our previous studies^3,27^.

### Epileptic patients

Altogether, 43 epileptic patients were included in this study. For the investigation of the cellular activity during physiological and epileptiform synchronies (experiments followed by cell clustering), tissue samples were obtained from 10 ResEpi patients (Suppl. Table 1), from frontal (n = 2 patients), temporal (n = 6), parietal (n = 1) and occipital (n = 1) lobes. These patients suffered from therapy resistant focal cortical epilepsy for 23.8 ± 16.6 years on average. The scalp EEG showed the presence of interictal spikes in all these patients. Three patients had temporal lobe epilepsy with hippocampal sclerosis; six epileptic patients were diagnosed with cortical dysgenesis, and three of them also suffered from hippocampal sclerosis. The remaining epileptic patient suffered from viral encephalitis. 5 females, 5 males, age range: 19–52 years, mean ± st.dev.: 31.9 ± 12.5 years. The intraoperative investigation of interictal activity involved 16 ResEpi, 6 TreatEpi and 4 NoMed patients, whereas in vivo chronic recordings were performed in 6 ResEpi patients. For more details on the age, gender, lobe, etiology and duration of epilepsy of epileptic patients see Suppl. Table 1.

### Non-epileptic patients

Altogether 23 NoEpi patients were included in this study. Tissue derived from 7 patients diagnosed with a brain tumour but without epilepsy was used for the acquisition of clustering data (NoEpi group, Suppl. Table 1). These patients—as stated in their anamnesis—did not suffer from any clinical manifestations of epileptic seizures before the date of their brain surgery. We obtained non-epileptic neocortical specimens from frontal (n = 1 patient), temporal (n = 3), parietal (n = 2) and occipital (n = 1) lobes. Five patients were diagnosed with glioblastoma, one with cavernoma and one with carcinoma metastasis. We received neocortical tissue either from the peritumoural area, or—if the surgical technique required—from a larger distance from the tumour. The distance of the resected sample from the tumour was provided by the neurosurgeon in each case, and the signs of a possible tumour infiltration of the obtained tissue was verified with post hoc anatomy in most of the cases (see in Suppl. Table 1). 5 females, 2 males, age range: 31–78 years, mean ± st.dev.: 62.1 ± 15.7 years. Sixteen NoEpi patients were included in the intraoperative recordings. For more details on the age, gender, lobe and etiology of these patients see Suppl. Table 1.

### Chronic intracortical recording

Twenty-four channel linear microelectrodes (ME, 150 µm distance between the contacts) were implanted into the neocortex of six ResEpi patients, below the clinical subdural grid electrodes (Fig. 1, Suppl. Fig. 1). Recordings were part of the clinical investigation aiming to identify the seizure focus and the eloquent areas prior to surgical therapy, and were performed as described earlier^58^. Briefly, continuous video-EEG observation was made for 5–7 days. ECoG from the clinical grid (20–48 channels, mastoid reference) was recorded concurrently with the patient video using the standard system of the hospital (acquisition rate: 400–5000 Hz/16 bit, band-pass: 0.1–200 Hz). The LFPg was simultaneously recorded in all layers of the neocortex with the intracortical ME. In three patients the LFPg was split into the EEG range (0.1–300 Hz) and single- and multiple-unit activity frequency (300–5000 Hz) as described in^58^. In the three other patients the LFPg was recorded using the same recording system as in the postoperative in vitro experiments (see later). In four cases (patients E29, E45, O37 and O39) the exact intracortical location of the ME was determined with post hoc anatomical examinations (see later).

### Intraoperative electrocorticography

To assess the epileptogenicity of the tissue samples derived from epileptic and—seemingly non-epileptic—tumour patients, we made intraoperative electrocorticography (ECoG) from 41 patients (16 ResEpi, 6 TreatEpi, 4 NoMed and 15 NoEpi patients). With the dura mater opened, we placed an 8-points clinical strip electrode (AD TECH Medical Instrument Corp., Racine, WI, USA) on the pial surface of the neocortical tissue to be resected and recorded for about 10–15 min with the standard hospital system (see above). We documented which electrode contact was above the obtained tissue sample and analysed how the presence of interictal spikes on the ECoG is related to the clinical manifestations of epilepsy (patient groups) as well as to the ability of the neocortex to generate SPA in vitro.

### Tissue preparation and in vitro recording

Tissue preparation and recording were performed as described previously^3^. Briefly, tissue was transported from the operating room to the laboratory (located in the same building) in a cold, oxygenated solution containing (in mM) 248 d-sucrose, 26 NaHCO_3_, 1 KCl, 1 CaCl_2_, 10 MgCl_2_, 10 d-glucose and 1 phenol red, equilibrated with 5% CO_2_ in 95% O_2_. Neocortical slices of 500 µm thickness were cut with a Leica VT1000S vibratome (RRID:SCR_016495). They were transferred and maintained at 35–37 °C in an interface chamber perfused with a standard physiological solution containing (in mM) 124 NaCl, 26 NaHCO_3_, 3.5 KCl, 1 MgCl_2_, 1 CaCl_2_, and 10 D-glucose, equilibrated with 5% CO_2_ in 95% O_2._

The local field potential gradient (LFPg) was recorded with a 24 contact (distance between contacts: 150 µm) laminar microelectrode^74^, and a custom-made voltage gradient amplifier of pass-band 0.01 Hz–10 kHz. Signals were digitized with a 32 channel, 16-bit resolution analogue-to-digital converter (National Instruments, Austin TX, USA) at 20 kHz sampling rate, recorded with a home written routine in LabView8.6 (National Instruments, Austin TX, USA, RRID:SCR_014325). The linear 24 channel microelectrode was placed perpendicular to the pial surface, and slices were mapped from one end to the other at every 300–400 µm. Usually channels 1–8 were in the supragranular, channels 9–13 in the granular and channels 14–23 were in the infragranular layers. Channel positions were determined according to the thickness of the neocortex of the given patient and corrected if necessary.

### Data analysis

SPA/eIED detection, LFPg and multiunit activity (MUA) were analyzed with the Neuroscan Edit4.5 program (Compumedics Neuroscan, Charlotte, NC, USA), as described in our previous study. The LFPg peaks of the SPAs/eIEDs were taken as time zero for further event-related analyses. The location of SPAs/eIEDs—supragranular, granular, infragranular—was determined in each case. Baseline correction (− 150 to − 50 ms) was applied to averaged LFPg and MUA^3^.

Single cells were clustered from records high-pass filtered at 500 Hz with either a home written program for Matlab (The MathWorks, Natick, MA, USA) or the program Klusters^75^. Only neurons with a clear refractory period of at least 1.5 ms were included. Action potentials (AP) of single cells detected on extracellular electrodes during epileptic seizures might be difficult because of the distorted waveform^76^ or the amplitude decrease^77^ of the APs. We did not face this problem during the spontaneously emerging in vitro interictal epileptic discharges (eIED). Furthermore, we were aware that AP waveform slightly changes during burst activity and clustered the APs accordingly. AP waveform analysis was performed in Matlab, on averaged APs (from wide-band recordings). Two independent criteria were used to separate principal cells (PC) and interneurons (IN), unbiased by the tissue of origin. The average duration of PC APs is significantly higher than that of INs^78^. The AP width was measured at the half of the largest amplitude (halfmax). The cell was considered to be PC if this value was larger than 0.4 ms, and IN if it was less than 0.2 ms. The other criterion was discharge dynamics^39^. A high peak at 3–10 ms followed by a fast exponential decay on the autocorrelogram was characteristic to “intrinsically bursting” PCs. If the peak was lacking but there was sustained firing, or the peak was > 10 ms, the cell was considered to be a regular firing PC. The remaining cells with halfmax amplitude of > 0.4 ms were categorized as PCs with unclear firing. A slow rise on the autocorrelogram together with a slow decay identified INs. Cells with AP widths between 0.2 and 0.4 ms and non-characteristic autocorrelogram were defined as unclassified cells (UC). In the intact hippocampus and neocortex, the firing frequency of the cells gives additional information on their identity: PCs have a significantly lower firing rate than INs^78^. In vitro procedures considerably alter the living conditions of the cells. Therefore, we did not consider this criterion for the identification of the cell type. The location of the single cells was determined in each recording, as described above (SPA location).

Analysis of single cell firing was investigated with a home written program in Matlab. For each cell, average firing frequency, inter-spike-interval (ISI, note that spike means action potential in this case), and a measure for burstiness (percentage of APs within bursts) were calculated. When a set of three APs was detected within 20 ms, they were considered to be part of a potential burst. Bursts containing more than three APs could be longer than 20 ms, but each group of three consecutive APs had to lie within a 20 ms period. Moreover, the first AP of the burst had to be preceded and the last AP had to be followed by a 20 ms silent period (modified from^20^).

The timing of single cell APs relative to the SPA/eIED was analysed using peri-event time histograms (PETHs) generated in two distinct ways, with a home written routine in Matlab. One PETH was generated for each cell-SPA/eIED comparison using an absolute (fixed) time period around the peak of the SPA/eIED (± 150 ms) with a bin size of 5 ms. The significance of the firing change was determined using a Monte Carlo approach. We calculated firing change quantiles by randomizing the inter-event intervals for SPA/eIED and cell APs and comparing the actual number of APs during the ± 50 ms time window around the SPA/eIED with those of 10,000 randomized event trains (for more details see “Supplementary material”). We calculated two further parameters describing how closely a cell was linked to SPA/eIED: dependency, defined as the proportion of APs falling within ± 50 ms around SPA/eIED events, and firing reliability, defined as the proportion of the same SPA/eIED time windows with at least one AP of the respective cell. As a second approach, the duration of the given SPA/eIED was taken into account when measuring the timing of cellular firing. We divided SPAs and eIEDs into five phases: “before”, “ascending”, “peak”, “descending” and “after”. For each of the five phases the significance of the firing change was determined for each cell-SPA/eIED pair, using the same randomization method as above (for more details see “Supplementary material”). Each cell was considered to fire preferably in the phase where it significantly increased its firing. If the firing was significantly increased in multiple phases, the phase with the highest firing frequency was chosen.

For investigating cellular interactions and the firing sequence of PCs and INs, pairwise cell–cell cross-correlograms were computed within each recording, using only APs occurring in the temporal vicinity of SPAs/eIEDs. The resulting cross-correlograms were tested for asymmetry using a binomial test and corrected for multiple testing due to the numerous cell–cell pairs. The cases of significant left–right asymmetry in the cross-correlogram were interpreted as one cell firing before the other. Two parameters were varied and tested repeatedly: the time range around the SPAs/eIEDs defining which cell APs were used (100 ms time windows, shifted by 50 ms around the LFPg peak for testing interactions at different stages of the SPA/eIED) and the time range in the resulting cross-correlogram being tested for asymmetry (20 ms time windows, shifted by 10 ms for testing interactions at different delays between the APs of the two cells). In case of multiple simultaneous population activities, the cell pairs were tested for each SPA/eIED type separately. Note that due to the design of the algorithm, only asymmetric cell–cell interactions were identified. Moreover, due to the time ranges investigated, the detected interactions do not necessarily reflect direct synaptic connections between the cells. In case of IN-PC pairs, we additionally checked the direction of the asymmetry, i.e. how often the IN vs. the PC was leading the firing.

### Statistics

As almost none of the data were normally distributed the non-parametric Mann–Whitney U-test was applied. As no two-way non-parametric tests are available to the best of our knowledge, in case of two-way tests (e.g. patient group and cell type), pairs of groups to be compared were tested and the resulting p-values corrected using the Bonferroni-Holm method. In addition to the p-value, the common language effect size (ES^79^) was calculated for each tested pair, due to the large variability in sample sizes of the tested groups. The ES is a sample size independent measure, ranging from 0.5 to 1. It describes how often a value randomly drawn from group A (the one with higher values) will be larger than a randomly drawn value from group B (the one with lower values).

In order to test for unequal proportions in contingency tables, the Chi-square test was applied. In case of significant outcomes, the standardised residuals ((observed-expected)/sqrt(expected)) were calculated post-hoc for each table cell to determine which cell(s) significantly deviate from their expected values.

## Supplementary Information

Supplementary Information.

## Data Availability

The data supporting the findings of this study are available on request from the corresponding author.

## Abbreviations

AP: Action potential
CSD: Current source density
ECoG: Electrocorticography
eIED: Experimental interictal-like epileptic discharge, emerging spontaneously in vitro
ES: Common language effect size
ISI: Inter-spike interval (spike = extracellularly detected action potential)
IB-PC: Intrinsically bursting principal cell
IED: Interictal epileptic discharge, recorded in vivo, in patients
IN: Interneuron
LFPg: Local field potential gradient
ME: Linear multielectrode
MUA: Multiple unit activity
NoEpi: Patients without preoperative epileptic seizures
NoMed: Patients with one (provoked) seizure who do not need medication
PC: Principal cell
ResEpi: Patients with pharmacoresistant epilepsy
PETH: Peri-event time histogram
RS-PC: Regularly spiking principal cell
SPA: Spontaneous synchronous population activity
SUA: Single unit activity
TreatEpi: Epileptic patients who are seizure free with appropriate medication
UC: Unclassified cell
