# Supplementary material for: Bursting of excitatory cells is linked to interictal epileptic discharge generation in humans
Source: Sci Rep. 2022 Apr 15;12:6280. doi: 10.1038/s41598-022-10319-4 (PMC9012754; doi:10.1038/s41598-022-10319-4)
Supplement: Supplementary file 1 — Supplementary Information. [file 41598_2022_10319_MOESM1_ESM.docx]

Bursting of excitatory cells is linked to interictal epileptic discharge generation in humans.

Katharina T. Hofer^1,2#^, Ágnes Kandrács^1,2^, Kinga Tóth^1^, Boglárka Hajnal^3,4^, Virág Bokodi^3^, Estilla Zsófia Tóth^1,4^, Loránd Erőss^3^, László Entz^3^, Attila G. Bagó^3^, Dániel Fabó^3^, István Ulbert^1,2,3+^ and Lucia Wittner^1,2,3+*^

1. Institute of Cognitive Neuroscience and Psychology, Research Center for Natural Sciences, Eötvös Loránd Research Network, 1117 Budapest, Hungary

2. Faculty of Information Technology and Bionics, Pázmány Péter Catholic University, 1083 Budapest, Hungary

3. National Institute of Mental Health, Neurology and Neurosurgery, 1143 Budapest, Hungary

4. Semmelweis University Doctoral School, 1026 Budapest, Hungary

# Present address: Department of Neurobiology, School of Medicine and Institute for Medical Research Israel-Canada, The Hebrew University, 91120 Jerusalem, Israel

+ Equally contributing authors

**Supplementary Data**

**Table of contents**

[1. Patient data 3](#_Toc97724857)

[2. In vivo correlates of in vitro SPA 6](#_Toc97724858)

[3. Epileptogenicity of the resected tissue 9](#_Toc97724859)

[4. Different types and properties of SPAs/eIEDs 14](#_Toc97724860)

[5. Cellular characteristics of clustered cells 16](#_Toc97724861)

[6. Discharge properties of neurons during SPA/eIED 18](#_Toc97724862)

[7. Temporal relationship between relative maximal firing of clustered neurons and SPA/eIED 20](#_Toc97724863)

[8. Relationship between the median ISI and the recurrence frequency 21](#_Toc97724864)

[9. Decrease in burstiness in the ResEpi compared to NoEpi tissue 23](#_Toc97724865)

[10. Quantification of cell firing during SPAs/eIEDs 24](#_Toc97724866)

[10.1. Motivation for the randomization algorithm 24](#_Toc97724867)

[10.2. Description of the randomization algorithm 24](#_Toc97724868)

[10.3. Dependence on the number of SPA/eIED and cell events 27](#_Toc97724869)

[10.4. Dependence on the chosen time window 30](#_Toc97724870)

[10.5. Relative time windows 31](#_Toc97724871)

[11. Local vs. non-local neurons 33](#_Toc97724872)

[12. Cell firing during multiple simultaneous SPA or SPA/eIED 34](#_Toc97724873)

[13. Hyperexcitability in ResEpi vs. NoEpi tissue 36](#_Toc97724874)

[14. References 39](#_Toc97724875)

# Patient data

**Supplementary Table 1. Patient data**

Pts: patients; (y)= years, IOP rec= intraoperative ECoG recording, Chronic rec: chronic in vivo ECoG & microelectrode recording; F=female, M=male, N/A=not available; Distance from tumour: close < 3 cm, distant > 3 cm; not applicable: anaesthesia not applicable, tissue samples were used for in vitro experiment

Seizure onset zone is given in case of chronic recordings and in vitro samples, spiking activity is given in case of IOP recordings.

| **Epilepsy stage** | **Pts** | **Experiment** | **Gen-der** | **Age (y)** | **Diagnosis** | **Resected cortical region** | **Duration of epilepsy** | **Seizure onset zone or spiking activity** | **Distance from tumour** | **Anatomy of obtained tissue** | **Anaesthesia** |
| --- | --- | --- | --- | --- | --- | --- | --- | --- | --- | --- | --- |
| ResEpi | E1 | clustering | F | 22 | encephalitis | parietal | 1 month | + |  | normal/cell loss | not applicable |
| ResEpi | E2 | clustering | F | 21 | focal cortical dysplasia with glioneural heterotopia | frontal | 2 years | + |  | dysgenetic | not applicable |
| ResEpi | E5 | clustering | F | 18 | focal cortical dysplasia II B | frontal | 5 years | + |  | dysgenetic | not applicable |
| ResEpi | E6 | clustering | M | 51 | hippocampal sclerosis | temporal | 50 years | + |  | normal | not applicable |
| ResEpi | E8 | clustering | F | 33 | focal cortical dysplasia II B | occipital | 31 years | - & + |  | normal & dysgenetic | not applicable |
| ResEpi | E10 | clustering | M | 21 | hippocampal sclerosis | temporal | 21 years | - |  | normal | not applicable |
| ResEpi | E13 | clustering | F | 53 | hippocampal sclerosis | temporal | 40 years | - |  | normal | not applicable |
| ResEpi | E15 | clustering | M | 35 | focal cortical dysplasia + hippocampal sclerosis | temporal | 34 years | + |  | normal | not applicable |
| ResEpi | E16 | clustering | M | 26 | subependymal gliosis (dysgenesis) + hippocampal sclerosis | temporal | 24 years | + |  | normal | not applicable |
| ResEpi | E17 | clustering | F | 38 | focal cortical dysplasia + hippocampal sclerosis | temporal | 31 years | + |  | normal | not applicable |
| ResEpi | E39 | IOP rec | F | 36 | hippocampal sclerosis | temporal | 30 years | - |  | N/A | Sevoflurane |
| ResEpi | E40 | IOP rec | M | 32 | anaplastic oligoastrocytoma grade III | frontal | 4 years | - | close | normal | Propofol |
| ResEpi | E51 | IOP rec | M | 29 | aspecific gliosis | temporal | 14 years | - |  | normal | Propofol |
| ResEpi | T53 | IOP rec | M | 36 | diffuse astrocytoma grade II, IDH-1 mutation, ATRX loss | temporal | 6 months | + | close | infiltrated | Propofol |
| ResEpi | E53 | IOP rec | M | 38 | hippocampal sclerosis + aspecific gliosis | temporal | 6 years | + |  | gliotic/cell loss | Propofol |
| ResEpi | E54 | IOP rec | F | 28 | aspecific gliosis | temporal | 19 years | + |  | normal | Propofol |
| ResEpi | E57 | IOP rec | F | 18 | focal cortical dysplasia II B with balloon cells | frontal | 15 years | + |  | dysgenetic | Propofol |
| ResEpi | E58 | IOP rec | F | 35 | haemangioma cavernosum | temporal | 5 years | - | close | normal | Propofol, Sevoflurane |
| ResEpi | E59 | IOP rec | M | 30 | focal cortical dysplasia II B with balloon cells | parietal | 27 years | + |  | normal | Propofol |
| ResEpi | E61 | IOP rec | M | 36 | cavernoma | temporal | 22 years | + | distant | normal | Propofol, Sevoflurane |
| ResEpi | E66 | IOP rec | F | 41 | hippocampal and temporal gliosis, microglia activation | temporal | 9 years | + |  | dysgenetic | Propofol |
| ResEpi | HP11 | IOP rec | M | 69 | ganglioglioma grade I | temporal | 46 years | + | close | infiltrated | Propofol |
| ResEpi | HP12 | IOP rec | M | 26 | hippocampal sclerosis | temporal | 13 years | + |  | normal | Propofol |
| ResEpi | HP13 | IOP rec | M | 35 | diffuse low grade glioneural tumour grade I | temporal | 9 years | + | close | infiltrated | Propofol |
| ResEpi | HP15 | IOP rec | F | 35 | cortical gliosis | temporal | 4 years | + |  | normal | Sevoflurane |
| ResEpi | HP20 | IOP rec | M | 42 | focal cortical dysplasia II B with balloon cells | frontal | 12 years | - |  | dysgenetic | Propofol |
| ResEpi | HP25 | IOP rec | M | 28 | focal cortical dysplasia II B with balloon cells | frontal | 22 years | + |  | normal | Propofol |
| ResEpi | O31 | Chronic rec | M | 26 | focal cortical dysplasia | frontal | 22 years | - |  | N/A | no anaesthesia |
| ResEpi | O37 | Chronic rec | M | 18 | focal cortical dysplasia | frontal | 10 years | + |  | normal | no anaesthesia |
| ResEpi | O39 | Chronic rec | F | 34 | focal cortical dysplasia I B | frontal | 23 years | + |  | normal | no anaesthesia |
| ResEpi | O104 | Chronic rec | F | 44 | sclerosis tuberosa | parietal | 27 years | - |  | N/A | no anaesthesia |
| ResEpi | E29 | Chronic rec | M | 18 | focal cortical dysplasia | parietal | 5 years | - |  | normal | no anaesthesia |
| ResEpi | E45 | Chronic rec | M | 48 | focal cortical dysplasia II B, sclerosis tuberosa | frontal | 39 years | + |  | dysgenetic | no anaesthesia |
| TreatEpi | E47 | IOP rec | M | 27 | glioblastoma, grade IV | frontal | 2 years | - | close | N/A | Propofol |
| TreatEpi | E50 | IOP rec | M | 27 | focal cortical dysplasia IA, microdysplasia | temporal | 5 years | - |  | dysgenetic | Propofol |
| TreatEpi | T55 | IOP rec | F | 62 | lung adenocarcinoma metastaticum | frontal | 32 years | + | close | infiltrated | Propofol |
| TreatEpi | HP19 | IOP rec | F | 61 | glioblastoma multiforme, grade IV | temporal | N/A | - | close | infiltrated | Propofol |
| TreatEpi | HP21 | IOP rec | F | 59 | diffuse astrocytoma grade III | temporal | 1 month | + | close | infiltrated | Propofol |
| TreatEpi | HP31 | IOP rec | F | 30 | ganglioglioma | temporal | 10 months | - | distant | normal | Propofol |
| NoMed | T50 | IOP rec | M | 63 | glioblastoma multiforme, grade IV | frontal | N/A  1 episode, disorientation | - | close | infiltrated | Propofol |
| NoMed | T59 | IOP rec | F | 52 | glioblastoma, grade IV | temporal | 1 month  memory disturbance | - | close | N/A | Propofol |
| NoMed | E60 | IOP rec | F | 36 | anaplastic astrocytoma, grade III (IDH1 mutation, ATRX loss) | frontal | N/A  1 generalized tonic-clonic seizure | - | close | infiltrated | Propofol |
| NoMed | HP10 | IOP rec | M | 64 | glioblastoma, grade IV | temporal | 3 months  memory disturbance, vertigo, aggravated speech | - | distant | infiltrated | Sevoflurane |
| NoEpi | T4 | clustering | F | 69 | glioblastoma multiforme | temporal |  |  | distant | normal | not applicable |
| NoEpi | T6 | clustering | M | 31 | cavernoma, haematoma intracerebralis acuta | frontal |  |  | distant | normal | not applicable |
| NoEpi | T7 | clustering | F | 58 | glioblastoma multiforme, meningitis | temporal |  |  | close | infiltrated | not applicable |
| NoEpi | T8 | clustering | F | 78 | glioblastoma multiforme, astrocytoma grade IV | temporal |  |  | distant | normal | not applicable |
| NoEpi | T11 | clustering | F | 57 | glioblastoma multiforme grade IV | occipital |  |  | distant | normal | not applicable |
| NoEpi | T17 | clustering | F | 74 | glioblastoma multiforme grade IV | parietal |  |  | distant | normal | not applicable |
| NoEpi | T18 | clustering | M | 68 | melanoma malignum metastaticum | parietal |  |  | distant | normal | not applicable |
| NoEpi | T39 | IOP rec | M | 37 | central neurocytoma grade II | frontal |  |  | distant | normal | Propofol |
| NoEpi | T42 | IOP rec | F | 58 | breast carcinoma metastaticum | frontal |  |  | close | N/A | Propofol |
| NoEpi | T43 | IOP rec | M | 73 | melanoma malignum metastaticum | temporal |  |  | close | N/A | Propofol |
| NoEpi | T47 | IOP rec | M | 74 | glioblastoma multiforme grade IV | frontal |  |  | close | N/A | Propofol |
| NoEpi | T49 | IOP rec | M | 76 | glioblastoma multiforme, grade IV | frontal |  |  | close | N/A | Propofol |
| NoEpi | T51 | IOP rec | M | 62 | glioblastoma multiforme, grade IV | temporal |  |  | close | N/A | Propofol |
| NoEpi | T56 | IOP rec | M | 64 | glioblastoma multiforme grade IV | parietal |  |  | close | normal | Propofol |
| NoEpi | T57 | IOP rec | F | 73 | glioblastoma multiforme grade IV | frontal |  |  | close | infiltrated | Propofol |
| NoEpi | T58 | IOP rec | M | 42 | glioblastoma multiforme grade IV | frontal |  |  | close | normal | Propofol |
| NoEpi | T61 | IOP rec | F | 40 | anaplastic astrocytoma grade III | parietal |  |  | distant | normal | Desflurane |
| NoEpi | T67 | IOP rec | F | 57 | glioblastoma multiforme grade IV | parietal |  |  | close | normal | Propofol |
| NoEpi | T68 | IOP rec | F | 72 | neuroendocrine carcinoma metastaticum, grade III | parietal |  |  | distant | normal | Propofol, Sevoflurane |
| NoEpi | T69 | IOP rec | M | 49 | anaplastic astrocytoma grade III | frontal |  |  | distant | N/A | Propofol |
| NoEpi | HP9 | IOP rec | F | 64 | sarcomatoid mesothelioma | temporal |  |  | distant | normal | Propofol |
| NoEpi | HP14 | IOP rec | F | 81 | lung adenocarcinoma metastaticum | frontal |  |  | close | infiltrated | Propofol |
| NoEpi | HP18 | IOP rec | M | 32 | anaplastic astrocytoma grade III | temporal |  |  | close | infiltrated | Propofol |

# In vivo correlates of in vitro SPA

We intended to know whether synchronous activity similar to SPA recorded in vitro emerges in the living brain. To assess this question, we analysed chronic in vivo intracortical recordings performed with a recording system similar to the one used for in vitro experiments.

Population bursts were observed in all examined patients (Supplementary Fig 1) as distinct waves extending to 7-23 recording channels. They were comparable to in vitro SPAs^1,2^ in LFPg amplitude, duration, and location. These in vivo population bursts were different from interictal spikes: the waveform did not show the typical spike and wave, the duration and the LFPg amplitude were considerably lower than for IEDs. In contrast to IEDs, population bursts were usually not detectable on the ECoG channel located at the lowest distance from the intracortical ME. In two cases with chronic intracortical recordings (Patients E29, shown on Fig. 1 and E45, shown on Supplementary Fig. 1), we made in vitro experiments on slices prepared from the resected tissue and recorded spontaneously emerging SPAs.

The question arises whether the population bursts seen with the intracortical microelectrode are physiological synchronies or are related to epileptic processes. They show similarities to phenomena recorded by Schevon and colleagues^3^, identified as microdischarges and considered to be epileptiform events. The different experimental settings make the comparison difficult. Schevon and colleagues^3^ used a 4x4 mm 96 channel microelectrode array recording only from neocortical layers II-III, parallel with the pial surface, whereas we used a linear multielectrode recording perpendicular to the pial surface, across all neocortical layers. Another concern is that they recorded the local field potential with referential recordings, whereas we detected the local field potential gradient. Thus, we cannot be sure that our population bursts correspond to the microdischarges described in Schevon *et al.*^3^

Our in vivo chronic experiments included only epileptic patients (similar to Schevon *et al.*^3^), therefore we cannot exclude the possibility that the recorded population events are related to epileptic processes. However, in three cases out of the six, the ME was located outside of the seizure focus, and SPA-like events were generated in the neocortex (Patients O39, E45, O104, Supplementary Fig. 1). This may suggest the physiological nature of the in vivo detected synchronous events, but since chronic intracortical recordings cannot be obtained from healthy subjects, the question remains opened.


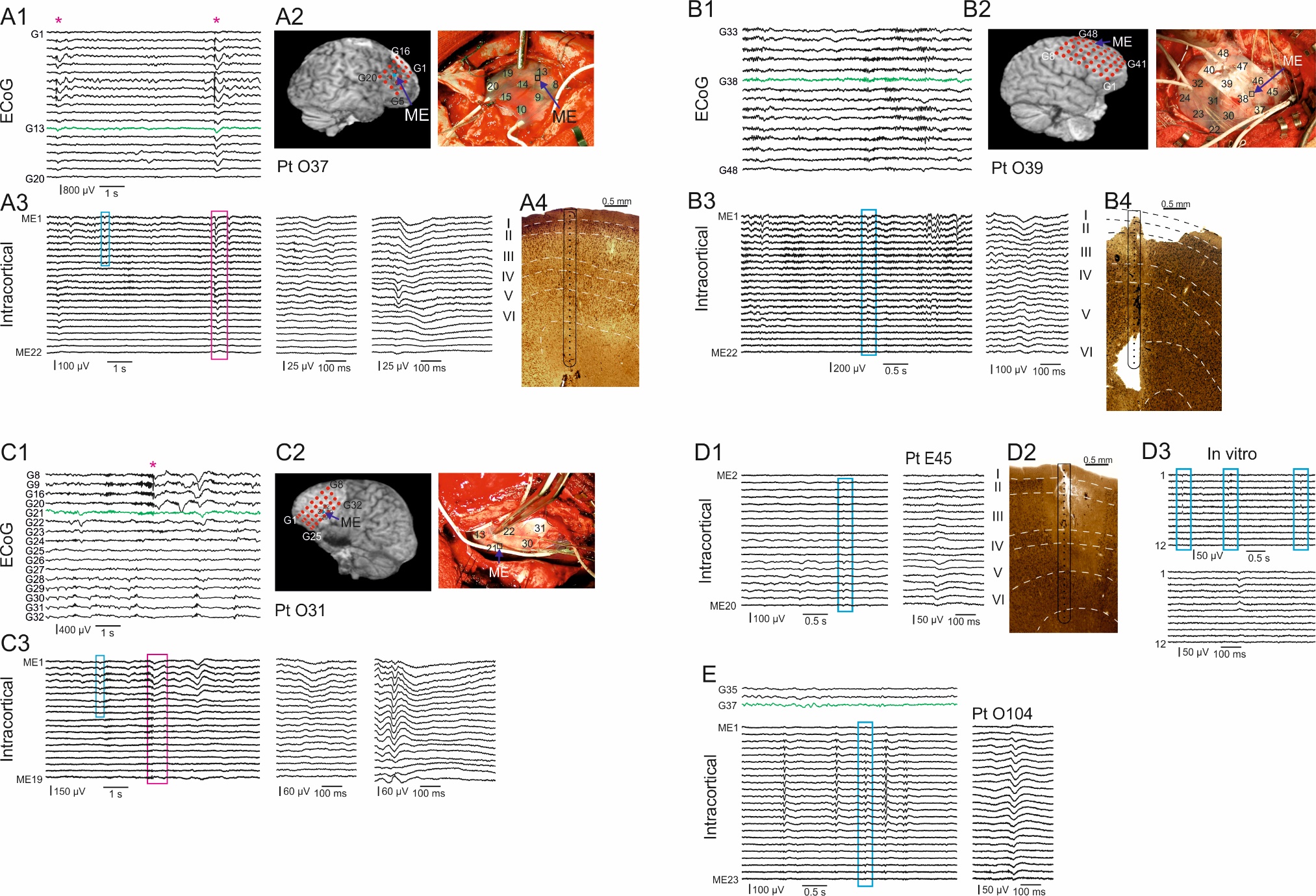


**Supplementary Figure 1. SPA recorded in vivo**

In vivo chronic intracortical recordings were made with the aid of a linear 24 channel microelectrode. Microelectrodes (ME) were implanted into the neocortex of 6 ResEpi patients, below the subdural grid electrodes (A2, B2, C2), as part of the preoperative clinical investigation of the patients. Grid electrodes recorded ECoG from the brain surface, while ME simultaneously recorded the intracortical LFPg. Post hoc anatomy (A4, B4, D2) demonstrated that the ME detected signals from all layers of the neocortex. Green channel on the ECoG (A1, B1, C1, E) marks the closest grid contact to the ME. Intracortical LFPg recordings showed the emergence of synchronous events similar to in vitro SPA in all examined patients (blue rectangle, magnified on middle panels on A3, C3, and on right panels on B3, D1, E). These synchronous events were different from interictal spikes (magenta asterisks on ECoG and magenta rectangles on intracortical recordings, the latters magnified on the right panel on A3, C3). Postoperative in vitro recordings showed that SPA was generated in slices derived from these patients (D3).

# Epileptogenicity of the resected tissue

Brain tumours are often related to epilepsy^4^ and the absence of preoperative clinical manifestations of epileptic seizures in tumour patients does not necessarily mean that they are not epileptic. Seizures can be overlooked, and the brain can produce subclinical epileptic activity, and thus, epilepsy or epileptic activity might remain unrecognised. Furthermore, although the presence of interictal spikes is one of the most important diagnostic tools for epilepsy, interictal spikes can be absent in the EEG recordings of epileptic patients, even though they suffer from seizures.

We observed interictal spiking activity in the intraoperative ECoG records in 15/16 ResEpi, in 3/6 TreatEpi, in 2/4 NoMed, whereas only in 5/15 NoEpi patients. Note that in most of the patients without preoperative epileptic seizures (NoEpi group) no interictal spikes were detected on the ECoG. The presence of IEDs in the NoEpi patients however might not result from the epileptogenicity of their tumour, as the most commonly used anaesthetics – such as sevoflurane^5,6^ and propofol^7^ – have proconvulsant effects. Forty of our 41 patients received either sevoflurane or propofol or both during their surgery (Supplementary Table 1). All five NoEpi patients showing IED activity were under propofol anaesthesia. This implies that we cannot be certain whether spiking activity of the NoEpi patients is an attribute of their state of health or is related to the anaesthetic used during the operation. Still, we have to note that the ECoG of most ResEpi patients displayed IED, whereas most NoEpi patients showed no spiking activity.

IEDs were observed only on a subset of the ECoG electrode contacts, in all patient groups. Therefore, we noted the spiking activity on the electrode contact located above the obtained tissue sample and related it to the presence of SPA in vitro (Supplementary Table 2, Supplementary Fig. 2). In one NoEpi case (T43) we obtained two specimens, one from a spiking, and the other from a non-spiking area. SPA emerged in most but not all human neocortical tissue samples (*n*=13/16 ResEpi, 5/6 TreatEpi, 1/4 NoMed and 12/15 NoEpi). To assess the differences related to epilepsy, and also due to their low numbers, we excluded TreatEpi and NoMed specimens, and made a comparison only between ResEpi and NoEpi samples. IEDs were observed in 10/13 ResEpi specimens generating SPA, whereas only 3/13 NoEpi samples with SPA showed spiking activity on the ECoG electrode located above the obtained tissue sample (significantly different, Fisher exact test, *P*<0.05). 10/11 ResEpi and all (3/3) NoEpi samples with intraoperative spiking activity (IED+) initiated SPA in postoperative experiments. Tissue samples derived from regions without IED generated SPA in 3/5 ResEpi and in 10/13 NoEpi specimens. Occasionally we obtained neocortical tissue which did not generate SPA (*n*=3 ResEpi and *n*=3 NoEpi samples).

In summary, SPA emerged in similar percentages of ResEpi and NoEpi samples, independently of the presence of IEDs in vivo. Numerous NoEpi specimens without the signs of interictal spiking activity in vivo were generating SPA (*n*=10). IEDs were not detected at all in the ECoG in seven out of these ten samples. The patient dataset used for the intraoperative ECoG recordings does not overlap with the patients used for the cellular firing properties during synchronies. Therefore, we cannot be certain about the epileptogenicity of the samples used for the clustering data, but our analysis on the relationship of IED and the presence of in vitro SPA shows, that SPA emerges in brain samples with and without in vivo spiking activity. The fact that SPA is generated in slices derived from NoEpi patients without the signs of IED strongly suggest that synchronous population activity seen in human slices can be considered as a physiological process.

**Supplementary Table 2.** **Relationship between intraoperative spiking activity and the presence of in vitro SPA.**

The table shows the number of patients with (IED+) or without (IED–) spiking activity above the tissue sample used for in vitro experiment, related to the emergence of SPA in vitro. Note that both samples from one NoEpi patient (T43, marked with *) with and without spiking activity on the ECoG generated SPA in vitro.

| Patient group | Total number of patients | IED+  SPA+ | IED–  SPA+ | IED+  SPA– | IED–  SPA– |
| --- | --- | --- | --- | --- | --- |
| ResEpi | 16 | *n*=10  E53, E57, E59, E61, E66, HP11, HP12, HP13, HP15, HP25 | *n*=3  E39, E40, E51 | *n*=1  T53 | *n*=2  E58, HP20 |
| TreatEpi | 6 | *n*=2  T55, HP21 | *n*=3  E50, HP19, HP31 | *n*=0 | *n*=1  E47 |
| NoMed | 4 | *n*=0 | *n*=1  T50 | *n*=0 | *n*=3  T59, E60, HP10 |
| NoEpi | 15 | *n*=3  T43*, T58, T67 | *n*=10  T39, T43*, T47, T56, T61, T68, T69, HP9, HP14, HP18 | *n*=0 | *n*=3  T42, T49, T51 |


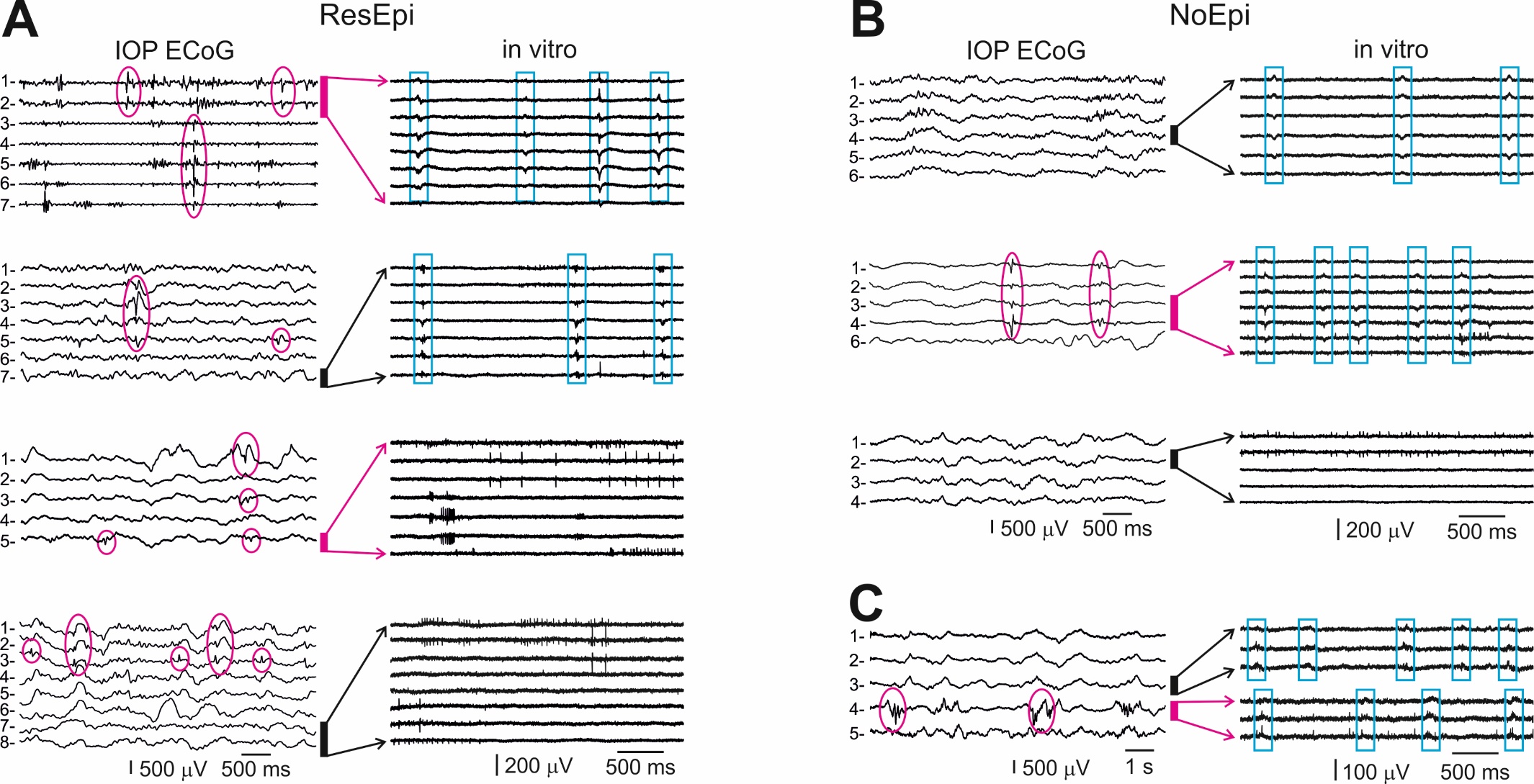


**Supplementary Figure 2. Relation between in vivo IED and in vitro SPA**

Intraoperative (IOP) ECoG recordings (left panels on A, B, C) confirmed the presence or absence of interictal spiking activity of the neocortical tissue used for in vitro experiments (right panels on A, B, C). In vitro SPA (blue rectangle) was generated independently of the presence of IEDs (magenta circles) on the ECoG. IED was detected in 15/16 ResEpi patients (A). The resected tissue derived from a non-spiking area (black line and arrows in the middle) in 5/16 cases, and from a spiking area (magenta line and arrows) in 11/16 cases. In contrast, in NoEpi patients (B, C) IEDs were seen in 5/15 patients, the in vitro examined specimen derived from a non-spiking area (black line and arrows) in 13/15 cases, and from a spiking area (magenta line and arrows) in 3/15 cases. Subfigure C shows the case where we obtained two specimens from the same patient, one from a non-spiking, one from a spiking area. Both tissue samples generated SPAs in vitro.

# Different types and properties of SPAs/eIEDs

Based on the location within the neocortex, we distinguished several types of SPAs.^2^ Altogether, in tissue from NoEpi patients we analysed 21 SPAs confined to the supragranular layer, 7 were in the supragranular+granular, 6 in the granular, 3 in the granular+infragranular and 5 in the infragranular layers. In the ResEpi group, we examined 24 SPAs located in the supragranular layer, 7 in the supragranular+granular, 6 in the granular, 3 in the granular+infragranular, 3 in the infragranular layers, and 1 SPA spread through the entire width of the neocortex. Ex vivo (experimental) IEDs (eIEDs) were detected in the granular (*n*=1), the granular+infragranular (*n*=4) and the infragranular layer (*n*=2). In 10/29 and 7/37 recordings simultaneous multiple SPAs were generated at one recording site, from NoEpi and ResEpi tissue, respectively. Furthermore, in five recordings where eIEDs were detected, additional SPAs emerged.

Results and significances should be treated cautiously in case of eIEDs, since the sample size of the eIED group (7 eIEDs in 7 recordings vs. 44 ResEpi SPAs in 35 recordings), as well as the numbers of eIED events/recording were considerably lower than those of ResEpi SPAs. Thus, to judge differences between groups, we also considered the common language effect sizes (ES) in addition to the *P*-values.

**Supplementary Table 3. Number and properties of analyzed SPAs and eIEDs.**

Results are given in median [1^st^ – 3^rd^ quartiles]. Results should be treated cautiously in case of eIEDs, since the sample size of the eIED group (7 eIEDs in 7 recordings vs. 44 ResEpi SPAs in 35 recordings), as well as the numbers of eIED events/recording were considerably lower than those of ResEpi SPAs. Thus, to judge differences between groups, we also considered the common language effect sizes (ES) in addition to the *P*-values.

|  | NoEpi SPA | ResEpi SPA | ResEpi eIED | Significances |
| --- | --- | --- | --- | --- |
| Number of patients | 7 | 10 | 4 |  |
| Number of slices | 21 | 31 | 5 |  |
| Number of recordings | 29 | 35 | 7 |  |
| Number of synchronous activities | 42 | 44 | 7 |  |
| Total number of synchronous events | 19 518 | 23 389 | 245 |  |
| Number of events/synchronous activity | 323.5 [177-677]  (range: 74-1744) | 461 [226.5-773.5]  (range: 37-1405) | 15 [9.5-43]  (range: 8-125) | ResEpi eIED < ResEpi SPA: p<0.005  ResEpi eIED < NoEpi SPA:  p<0.005 |
| Number of synchronous events/slice | 523 [191-1 110]  (range: 83-2 463) | 599 [248.5-967.5]  (range: 58-2 191) | 26 [8-63]  (range: 8-140) | ResEpi eIED < ResEpi SPA: p<0.005  ResEpi eIED < NoEpi SPA:  p<0.005 |
| Total number of synchronous events/lobes  - frontal  - temporal  - occipital  - parietal | 6 994  6 945  950  4 629 | 1 877  18 797  1 544  1 171 | 219  26 |  |
| Recurrence frequency (Hz) | 1.06  [0.76 - 1.48] | 1.03  [0.72 - 1.58] | 0.05  [0.03 - 0.13] | ResEpi eIED < ResEpi SPA: p<0.0001, ES=0.98 |
| Local field potential gradient (µV) | 15.41  [10.25 - 23.71] | 23.22  [17.02 - 30.29] | 74.36  [63.10 - 92.62] | NoEpi SPA < ResEpi SPA: p<0.01, ES=0.69; ResEpi SPA < ResEpi eIED: p<0.001, ES=0.95 |
| Multiple unit activity (µV) | 0.90  [0.52 - 1.49] | 1.06  [0.70 - 2.03] | 5.81  [3.53 - 8.68] | ResEpi SPA < ResEpi eIED: p<0.01, ES=0.85 |

# Cellular characteristics of clustered cells

**Supplementary Table 4.** Cellular characteristics of clustered neurons (median [1^st^ and 3^rd^ quartile] (mean±SD))

|  |  | Number of clustered cells | Firing frequency (Hz) | Interevent interval (s) | Firing irregularity (interevent interval variability) | Burstiness |
| --- | --- | --- | --- | --- | --- | --- |
| NoEpi | **Total** | **406** | **0.2 [0.2 - 0.5]**  **(0.8 ± 1.7)** | **0.1 [0.1 – 1.0]**  **(5.0 ± 24.5)** | **5.5 [2.3 - 11.3]**  **(11.8 ± 23.4)** | **0.00 [0.00 - 6.00]**  **(6.58 ± 14.43)** |
|  | Principal cells | 132 (32.5%) | 0.2 [0.1 - 0.5]  (0.9 ± 2.0) | 0.1 [0.0 - 0.2]  (2.5 ± 12.8) | 8.0 [4.4 - 14.8]  (14.6 ± 22.4) | 1.81 [0.00 - 12.29]  (10.07 ± 18.00) |
|  | Interneurons | 104 (25.6%) | 0.2 [0.1 - 0.9]  (1.0 ± 1.9) | 0.4 [0.1 - 2.6]  (6.9 ± 35.7) | 2.7 [1.2 - 7.3]  (12.2 ± 30.6) | 0.00 [0.00 - 1.58]  (2.46 ± 6.70) |
|  | Unclassified cells | 170 (41.9%) | 0.2 [0.1 - 0.4]  (0.5 ± 1.2) | 0.1 [0.1 - 1.3]  (5.9 ± 23.0) | 5.1 [2.6 - 10.2]  (9.5 ± 18.5) | 0.00 [0.00 - 5.71]  (6.41 ± 14.16) |
| ResEpi | **Total** | **351** | **0.4 [0.2 - 0.8]**  **(0.9 ± 1.9)** | **0.3 [0.1 - 1.1]**  **(1.7 ± 7.2)** | **3.7 [1.7 - 8.8]**  **(7.5 ± 10.8)** | **0.00 [0.00 - 0.96]**  **(2.45 ± 7.81)** |
|  | Principal cells | 119 (33.9%) | 0.4 [0.2 - 0.8]  (1.0 ± 1.6) | 0.2 [0.1 - 0.4]  (0.7 ± 1.8) | 6.3 [2.4 - 12.8]  (9.9 ± 10.9) | 0.00 [0.00 - 1.99]  (2.82 ± 8.52) |
|  | Interneurons | 102 (29.1%) | 0.6 [0.2 - 1.1]  (1.3 ± 2.6) | 0.5 [0.2 - 1.1]  (2.2 ± 10.0) | 2.4 [1.4 - 4.3]  (5.3 ± 8.2) | 0.00 [0.00 - 0.00]  (1.59 ± 4.99) |
|  | Unclassified cells | 130 (37.0%) | 0.3 [0.1 - 0.6]  (0.6 ± 1.2) | 0.4 [0.1 - 1.7]  (2.2 ± 7.6) | 3.5 [1.9 - 7.5]  (7.0 ± 12.1) | 0.00 [0.00 - 0.61]  (2.78 ± 8.87) |
| Significant differences |  |  | NoEpi total < ResEpi total: p<1^-09^, ES=0.63  NoEpi UC < NoEpi IN: p<0.05, ES=0.59  ResEpi UC < ResEpi PC: p<0.05, ES=0.60  ResEpi UC < ResEpi IN: p<0.001, ES=0.65  NoEpi PC < ResEpi PC: p<0.001, ES=0.65  NoEpi IN < ResEpi IN: p<0.05, ES=0.62  NoEpi UC < ResEpi UC: p<0.01, ES=0.61 | NoEpi total < ResEpi total: p<10^-05^, ES=0.59  NoEpi PC < NoEpi IN: p<10^-05^, ES=0.70  NoEpi PC < NoEpi UC: p<0.01, ES=0.61  ResEpi PC < ResEpi IN: p<0.001, ES=0.66  ResEpi PC < ResEpi UC: p<0.01, ES=0.64  NoEpi PC < ResEpi PC: p<0.001, ES=0.65  NoEpi UC < ResEpi UC: p<0.01, ES=0.61 | ResEpi total < NoEpi total: p<0.001, ES=0.57  NoEpi IN < NoEpi PC: p<10^-06^, ES=0.71  NoEpi UC < NoEpi PC: p<0.01, ES=0.63  NoEpi IN < NoEpi UC: p<0.01, ES=0.62  ResEpi IN < ResEpi PC: p<10^-05^, ES=0.71  ResEpi UC < ResEpi PC: p<0.01, ES=0.62  ResEpi IN < ResEpi UC: p<0.05, ES=0.60  ResEpi UC < NoEpi UC: p<0.05, ES=0.58 | ResEpi total < NoEpi total: p<1e-06, ES=0.59  NoEpi IN < NoEpi PC: p<0.001, ES=0.65  NoEpi UC < NoEpi PC: p<0.05, ES=0.59  PC ResEpi < PC NoEpi: p<0.0001, ES=0.65  UC ResEpi < UC NoEpi: p<0.05, ES=0.58 |

PC types: NoEpi n=132, IB: 15.2%, RS: 35.6%, unclear firing: 49.2% and ResEpi n=119, IB: 10.1%, RS: 44.5%, unclear firing: 45.4%

IB: intrinsically bursting, RS: regular spiking firing pattern on the autocorrelogram

Neurons responding with increased firing to SPAs had a burstiness index similar to that of decreased and unchanged cells (NoEpi SPA, increased cells (*n*=131): 0.0 [0.0-0.1] (0.09±0.2), non-increased cells (*n*=472): 0.0 [0.0-0.1] (0.07±0.15), *P*>0.8, ES=0.50; ResEpi SPA, increased cells (*n*=151): 0.0 [0.0-0.0] (0.02±0.08), non-increased cells (*n*=209): 0.0 [0.0-0.0] (0.02±0.06), *P*>0.1, ES=0.54). In contrast, neurons with increased firing rates during eIEDs (*n*=52) showed a significantly higher burstiness 0.0 [0.0-0.1] (0.07±0.15), than non-increased cells (*n*=89): 0.0 [0.0-0.0] (0.01±0.03), *P*<0.05, ES=0.60.

# Discharge properties of neurons during SPA/eIED

**Supplementary Table 5.** Discharge properties of neurons during SPA/eIED (median [1^st^ and 3^rd^ quartile] (mean±SD))

Significantly higher proportion of neurons fire during ResEpi SPA than NoEpi SPA. The firing rate change quantile (an index for firing increase, see later) is higher for cells during ResEpi SPA, than during NoEpi SPA. INs have higher firing change quantile than PCs, both in NoEpi and ResEpi SPA. Both the reliability and dependency of neurons to population activities were higher for ResEpi SPA than for NoEpi SPA.

|  |  | Increased firing rate (n) | Unchanged firing rate (n) | Decreased firing rate (n) | Firing rate change quantile during SPA/eIED | Reliability  (% of SPA/eIEDs with cell action potentials) | Dependency  (% of cell action potentials during SPA/eIED) | Time of maximal firing of increased cells relative to the peak of the SPA/eIED (ms) |
| --- | --- | --- | --- | --- | --- | --- | --- | --- |
| **NoEpi SPA** | **Total (603)** | **131**  **(21.7%)** | **457**  **(75.8%)** | **15**  **(2.5%)** | **0.64 [0.34 - 0.95]**  **(0.62 ± 0.32)** | **1.3 [0.3 - 6.3]**  **(7.9 ± 16.7)** | **13.4 [5.9 - 25.0]**  **(19.2 ± 20.5)** | **0 [-5 - 10]**  **(-0.5 ± 66.4)** |
|  | Principal cells (301) | 32  (14.7%) | 178  (82.0%) | 7  (3.2%) | 0.55 [0.33 - 0.89]  (0.57 ± 0.32) | 1.1 [0.3 - 4.6]  (7.2 ± 15.9) | 12.5 [5.5 - 23.3]  (16.0 ± 15.5) | 0 [-10 - 7.5]  (-12.0 ± 84.2) |
|  | Interneurons (113) | 47  (32.6%) | 96  (66.7%) | 1  (0.7%) | 0.73 [0.34 - 1.00]  (0.65 ± 0.33) | 2.2 [0.5 - 10.3]  (12.1 ± 22.7) | 13.7 [6.4 - 27.0]  (22.8 ± 25.2) | 0 [-3.8 - 8.8]  (-0.6 ± 65.7) |
|  | Unclassified cells (189) | 52  (21.5%) | 183  (75.6%) | 7  (2.9%) | 0.67 [0.37 - 0.95]  (0.64 ± 0.31) | 1.3 [0.2 - 5.2]  (6.1 ± 12.4) | 13.6 [5.9 - 25.8]  (20.0 ± 21.1) | 0 [-10 - 27.5]  (6.8 ± 53.8) |
| **ResEpi SPA** | **Total (360)** | **151**  **(41.9%)** | **195**  **(54.1%)** | **14**  **(3.9%)** | **0.92 [0.53 - 1.00]**  **(0.74 ± 0.32)** | **4.4 [1.8 - 15.8]**  **(12.7 ± 18.3)** | **18.8 [9.2 - 34.2]**  **(26.6 ± 23.3)** | **0 [-10 - 10]**  **(0.1 ± 38.2)** |
|  | Principal cells (158) | 33  (27.5%) | 81  (67.5%) | 6  (5.0%) | 0.76 [0.47 - 0.99]  (0.69 ± 0.32) | 3.8 [1.7 - 12.1]  (11.6 ± 18.3) | 15.2 [9.2 - 28.3]  (20.7 ± 16.8) | 0 [-10 - 5]  (3.9 ± 34.8) |
|  | Interneurons (100) | 61  (55.5%) | 47  (42.7%) | 2  (1.8%) | 1.00 [0.56 - 1.00]  (0.79 ± 0.30) | 10.9 [3.0 - 23.6]  (18.1 ± 20.5) | 21.1 [9.7 – 49.0]  (31.7 ± 27.0) | 0 [-5 - 10]  (5.7 ± 27.9) |
|  | Unclassified cells (96) | 57  (43.8%) | 67  (51.5%) | 6  (4.6%) | 0.95 [0.53 - 1.00]  (0.73 ± 0.34) | 3.2 [1.7 - 9.3]  (9.3 ± 15.2) | 19.5 [8.6 - 39.4]  (27.9 ± 24.1) | 0 [-10 - 6.3]  (-8.2 ± 47.6) |
| **ResEpi eIED** | **Total (141)** | **52**  **(36.9%)** | **89**  **(63.1%)** | **0**  **(0.0%)** | **0.81 [0.44 - 1.00]**  **(0.71 ± 0.29)** | **6.7 [0.0 - 27.1]**  **(18.9 ± 27.0)** | **1.8 [0.0 - 6.3]**  **(9.1 ± 18.3)** | **0 [-10 - 7.5]**  **(3.2 ± 30.9)** |
|  | Principal cells (42) | 14  (33.3%) | 28  (66.7%) | 0  (0.0%) | 0.83 [0.42 - 1.00]  (0.71 ± 0.29) | 6.4 [0.0 - 25.0]  (17.3 ± 26.3) | 1.9 [0.0 - 6.3]  (8.3 ± 17.2) | -10 [-15 - 0]  (-9.3 ± 14.9) |
|  | Interneurons (42) | 17  (40.5%) | 25  (59.5%) | 0  (0.0%) | 0.78 [0.49 - 1.00]  (0.73 ± 0.29) | 7.7 [0.0 - 40.0]  (22.3 ± 28.3) | 1.9 [0.0 - 6.3]  (7.7 ± 15.8) | 0 [-16.3 - 21.3]  (6.8 ± 45.7) |
|  | Unclassified cells (57) | 21  (36.8%) | 36  (63.2%) | 0  (0.0%) | 0.85 [0.43 - 1.00]  (0.70 ± 0.30) | 4.1 [0.0 - 21.4]  (17.6 ± 26.7) | 1.7 [0.0 - 8.2]  (10.7 ± 20.8) | 5 [-5 - 11.3]  (8.6 ± 21.7) |
| Significant differences |  | NoEpi SPA vs ResEpi SPA: p<10^-10^ (NoEpi increased < ResEpi increased and NoEpi unchanged > ResEpi unchanged)  ResEpi SPA vs ResEpi eIED: p<0.05  (SPA decreased > eIED decreased)  Considering PC-IN:  NoEpi SPA vs ResEpi SPA: p<10^-14^  ResEpi SPA vs ResEpi eIED: p<0.01 | | | NoEpi SPA total < ResEpi SPA total: p<10^-10^, ES=0.63  ResEpi SPA PC < ResEpi SPA IN: p<0.01, ES=0.64  PC NoEpi SPA < PC ResEpi SPA: p<0.01, ES=0.61  IN NoEpi SPA < IN ResEpi SPA: p<0.001, ES=0.65  UC NoEpi SPA < UC ResEpi SPA: p<0.01, ES=0.62 | NoEpi SPA total < ResEpi SPA total: p<10^-19^, ES=0.68  NoEpi SPA PC < NoEpi SPA IN: p<0.05, ES=0.59  ResEpi SPA PC < ResEpi SPA IN: p<0.05, ES=0.62  ResEpi SPA UC < ResEpi SPA IN: p<0.001, ES=0.65  PC NoEpi SPA < PC ResEpi SPA: p<10^-08^, ES=0.71  IN NoEpi SPA < IN ResEpi SPA: p<0.0001, ES=0.67  UC NoEpi SPA < UC ResEpi SPA: p<10^-05^, ES=0.66 | NoEpi SPA total < ResEpi SPA total: p<10^-08^, ES=0.61  ResEpi eIED total < ResEpi SPA total: p<10^-30^, ES=0.83  PC ResEpi eIED < PC ResEpi SPA: p<10^-09^, ES=0.84  PC NoEpi SPA < PC ResEpi SPA: p<0.05, ES=0.60  IN ResEpi eIED < IN ResEpi SPA: p<10^-10^, ES=0.86  IN NoEpi SPA < IN ResEpi SPA: p<0.05, ES=0.62  UC ResEpi eIED < UC ResEpi SPA: p<10^-09^, ES=0.81  UC NoEpi SPA < UC ResEpi SPA: p<0.01, ES=0.61 | n.s. |

# Temporal relationship between relative maximal firing of clustered neurons and SPA/eIED

**Supplementary Table 6.** Temporal relationship between maximal firing of clustered neurons and the relative phases of SPA/eIED

|  | NoEpi SPA | | | | ResEpi SPA | | | | ResEpi eIED | | | |
| --- | --- | --- | --- | --- | --- | --- | --- | --- | --- | --- | --- | --- |
|  | **Total number of cells n (%)** | PC | IN | UC | **Total number of cells n (%)** | PC | IN | UC | **Total number of cells n (%)** | PC | IN | UC |
| Before | **18 (3.0%)** | 8 (3.7%) | 1 (0.7%) | 9 (3.7%) | **9 (2.5%)** | 0 (0.0%) | 4 (3.6%) | 5 (3.8%) | **4 (2.8%)** | 0 (0.0%) | 1 (2.4%) | 3 (5.3%) |
| Ascending | **45 (7.5%)** | 11 (5.1%) | 13 (9.0%) | 21 (8.7%) | **38 (10.6%)** | 12 (10.0%) | 13 (11.8%) | 13 (10.0%) | **22 (15.6%)** | 9 (21.4%) | 7 (16.7%) | 6 (10.5%) |
| Peak | **71 (11.8%)** | 17 (7.8%) | 33 (22.9%) | 21 (8.7%) | **101 (28.1%)** | 23 (19.2%) | 39 (35.5%) | 39 (30.0%) | **28 (19.9%)** | 6 (14.3%) | 6 (14.3%) | 16 (28.1%) |
| Descending | **36 (6.0%)** | 9 (4.1%) | 7 (4.9%) | 20 (8.3%) | **29 (8.1%)** | 8 (6.7%) | 12 (10.9%) | 9 (6.9%) | **7 (5.0%)** | 1 (2.4%) | 4 (9.5%) | 2 (3.5%) |
| After | **28 (4.6%)** | 13 (6.0%) | 4 (2.8%) | 11 (4.5%) | **13 (3.6%)** | 3 (2.5%) | 4 (3.6%) | 6 (4.6%) | **5 (3.5%)** | 2 (4.8%) | 2 (4.8%) | 1 (1.8%) |
| **Total number of cells with increased firing** | **198**  **(32.8%)** | **58**  **(26.7%)** | **58**  **(40.3%)** | **82**  **(33.9%)** | **190**  **(52.8%)** | **46**  **(38.3%)** | **72**  **(65.5%)** | **72**  **(55.4%)** | **66**  **(46.8%)** | **18**  **(42.9%)** | **20**  **(47.6%)** | **28**  **(49.1%)** |
| **Total number of clustered cells** | **603** | **217** | **144** | **242** | **360** | **120** | **110** | **130** | **141** | **42** | **42** | **57** |

# Relationship between the median ISI and the recurrence frequency

In the main text, we describe an apparent contradiction: for some groups of cells, both the mean recurrence frequency as well as the median inter spike interval (ISI, where spike= action potential of the detected cells) are smaller in one group than the other, although these two parameters are expected to be inversely correlated with each other. The main problem is the multimodal and strongly skewed distributions the ISIs follow, making single number descriptions of these distributions (mean, median, etc) difficult to impossible. For simplicity, we will discuss the differences of the ISI median with the ISI mean, rather than with the mean recurrence frequency. The latter two are (except for rounding errors) perfect inverses of each other. This way, we can directly compare the mean and median of an ISI distribution and avoid having to compare seconds to Hertz.

Thus, the question becomes how it can be that the ISI median value is lower, and the ISI mean is higher in one group vs the other. Supplementary Fig. 3A shows an example of such an apparently contradicting case, comparing the ISI distributions of NoEpi PCs and ResEpi PCs (median ISI: NoEpi PC < ResEpi PC, *P*<0.001, *ES*=0.65; mean ISI: ResEpi PC < NoEpi PC, *P*<0.001, *ES*=0.65). Although both ISI distributions are strongly skewed, the tail of the NoEpi PC's ISI distribution is much thicker compared to ResEpi, reflecting a larger number of long ISIs. As the mean is very sensitive to such extreme values, the thick tail contributes to a high mean ISI. On the other hand, the larger number of very short ISIs of the NoEpi PCs contribute to a lower ISI median compared to ResEpi PCs. This indicates that NoEpi PCs fire more irregularly (more very short and very long ISIs) than ResEpi PCs, which fire more regularly (more middle length ISIs). Thus, the opposing results of median ISI and mean ISI (and thus recurrence frequency) do not constitute a contradiction but reflect differences in firing patterns.


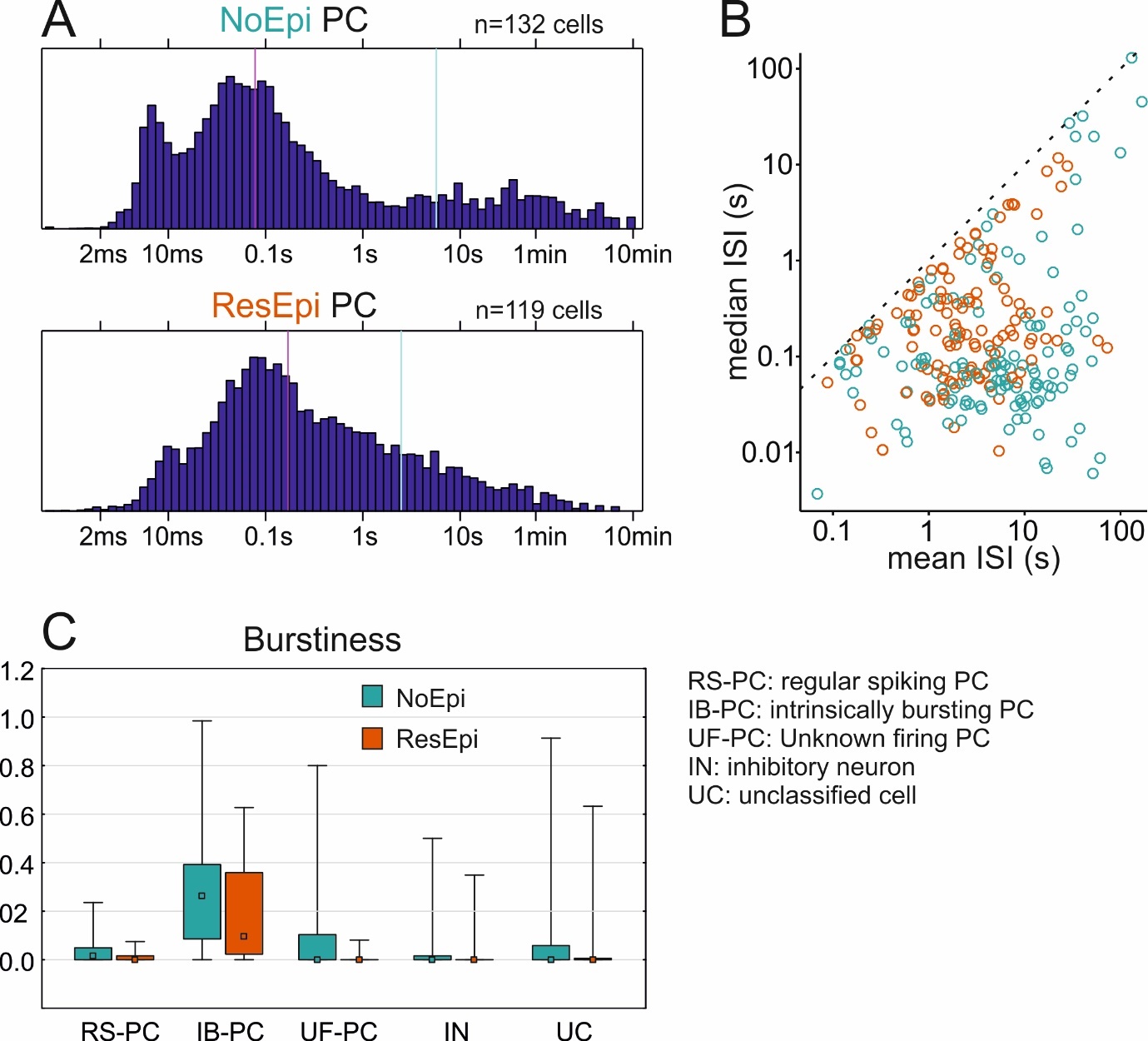


**Supplementary Figure 3. Discrepancy between mean and median ISI**

A: Mean ISI histograms (each cell was given the same weight) for NoEpi PCs and ResEpi PCs. The x-axis is shown on a logarithmic scale. Thus, the distribution of ISIs is more strongly skewed than it appears in the histogram. The y-axes were adjusted to normalize the histograms to the same area. Note that NoEpi has more very short (<10ms) and very long (>1min) ISIs compared to ResEpi, whereas ResEpi has more middle range (~1s) ISIs. The median (across cells) of the median ISI (magenta) and mean ISI (cyan) are indicated.

B: scatter plot of the mean ISI vs. the median ISI for each NoEpi PC (green) and ResEpi PC (orange). The dashed line indicates where the mean and median ISI are identical. The more irregular the cell firing (e.g., many short and some long ISIs), the larger the difference between mean and median ISI and the further away from the dashed line the cell gets plotted.

This difference in firing patterns can also be observed when directly comparing the median ISI with the mean ISI for each cell (Supplementary Fig. 3B). For a cell which fires perfectly regularly (i.e. all events are equally spaced and all ISIs are identical), the median and mean ISI would be equal and the cell is plotted along the dashed line in Supplementary Fig. 3B. However, another cell, comprising multiple short and a few long ISIs (resulting in the same mean ISI as the first cell), would have a shorter median ISI than the first cell and gets plotted below the dashed line. Thus, the distance from the dashed line reflects irregularity of cell firing. Note that irregular firing, in this context, is related to the asymmetry of the ISI distribution, not the magnitude of the ISI standard deviation. Supplementary Fig. 3B shows that NoEpi PCs tend to display a larger distance from the dashed line than ResEpi PCs and thus show a stronger discrepancy between mean and median ISI, indicating more irregular firing in the NoEpi group.

We can only speculate whether this is the consequence of a dynamic change in their firing properties related to epilepsy or the sign of cell death in epileptic tissue^2^ disproportionally affecting PCs firing in bursts.

# Decrease in burstiness in the ResEpi compared to NoEpi tissue

There is a striking difference in the short ISIs (<10 ms) between NoEpi and ResEpi PCs, falling in the range of the ISIs being within bursts (Supplementary Figure 3A). The decrease of APs within bursts is also reflected in the synaptically driven burstiness of the PCs in ResEpi (0.00 [0.00 - 1.99]), compared to NoEpi slices (1.81 [0.00 - 12.29], p<10^-4^, ES=0.65, Supplementary Table 4). These values include every PC, irrespective of its firing pattern (i.e., being regular spiking, RS-PC, intrinsically bursting, IB-PC or unknown firing, UF-PC). We analysed which PC types account for this change and found that the burstiness of RS-PC and UF-PC has significantly decreased in epileptic compared to non-epileptic tissue (Supplementary Table 7, p<0.005, Mann-Whitney U test). Contrary to our expectations, the burstiness of the IB-PCs was not significantly diminished (p=0.267, Supplementary Figure 4). Note that in case of UF-PCs the firing characteristics could not be determined, and this category might also contain RS-PCs and IB-PCs. The burstiness of unclassified cells (UC) was also significantly lower in ResEpi vs. in NoEpi slices, whereas interneurons (IN) did not change their burstiness in epilepsy. We also have to note that the group of UC possibly contains both PCs and INs.

**Supplementary Table 7.**

The burstiness index of the different cell types in ResEpi vs. NoEpi slices. Mann-Whitney U test, * p<0.005

| Burstiness | n | NoEpi | n | ResEpi |
| --- | --- | --- | --- | --- |
| RS-PC | 46 | 0.016 [0 – 0.049] | 53 | 0 [0 – 0.016] * |
| IB-PC | 20 | 0.261 [0.086 – 0.392] | 12 | 0.094 [0.023 – 0.359] n.s. |
| UF-PC | 65 | 0 [0 – 0.103] | 54 | 0 [0 – 0] * |
| IN | 104 | 0 [0 – 0.016] | 102 | 0 [0 – 0] n.s. |
| UC | 171 | 0 [0 – 0.058] | 130 | 0 [0 – 0.006] * |


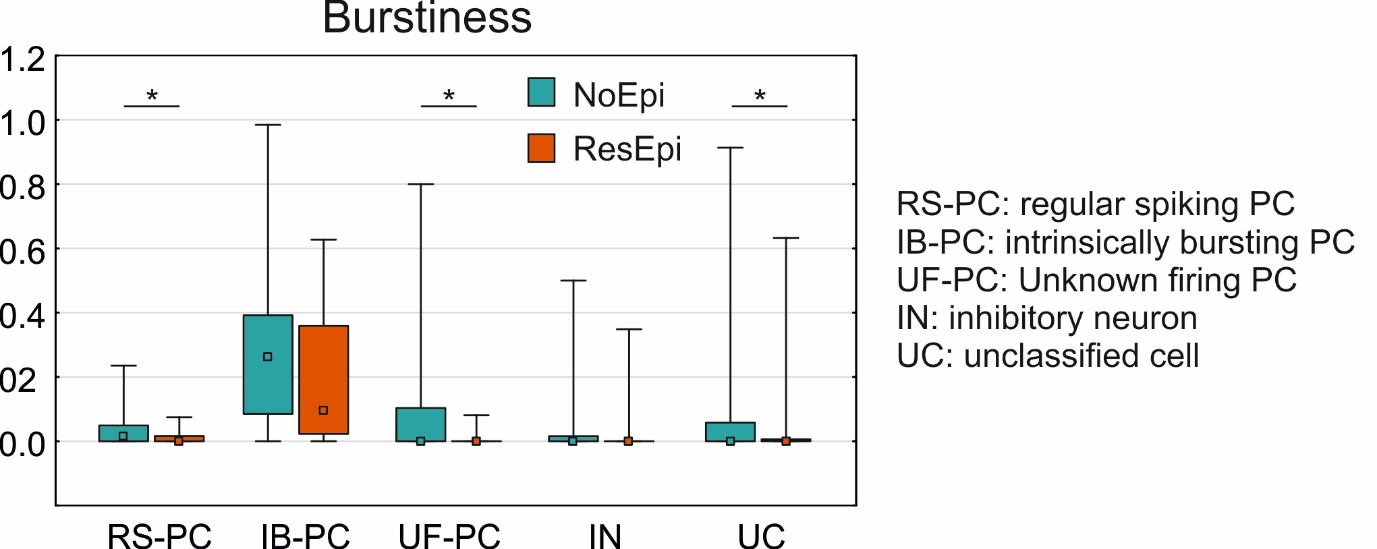


**Supplementary Figure 4. Burstiness of the different PC cell types**

The loss of the short (<10 ms) ISIs is reflected in the lower burstiness index of the PCs in the ResEpi compared to the NoEpi tissue. The burstiness index of RS-PC, UF-PC and UCs was significantly lower in ResEpi than in NoEpi slices (p<0.005). Median and 1^st^ – 3^rd^ quartiles are shown with maximal values.

# Quantification of cell firing during SPAs/eIEDs

## Motivation for the randomization algorithm

For each combination of one SPA/eIED and one cell, the firing of the latter was assessed in relation to the SPA/eIED. A straightforward approach for quantifying the firing change would be to calculate the firing frequency for the time window ±50 ms around the SPA/eIED, standardised to the baseline firing frequency. However, for cells with very low baseline firing, in cases where none of the action potentials (APs) fell into the time periods around the SPA/eIED, this calculation falsely implies strongly reduced firing during the SPA/eIED. To avoid wrongly identifying these cells as exhibiting decreased firing during the SPA/eIED, the significance (rather than the degree) of the firing change needed to be assessed. This was done using a Monte Carlo approach. Note that this analysis was performed for each single combination of cells and SPA/eIED (provided they originate from the same recording). Thus, a cell can display different responses to simultaneously occurring SPAs/eIEDs.

## Description of the randomization algorithm

Supplementary Fig. 5 shows how shuffled event trains were generated by randomly permuting the interevent intervals (ISIs) of both the cell and the SPA/eIED. Importantly, this process preserves many of the cell’s and the SPA/eIED’s recurrence properties such as e.g. the median ISI, the mean recurrence frequency and the ISI variability. However, any potentially existing temporal relationship between the cell firing and the SPA/eIED is lost during randomization.


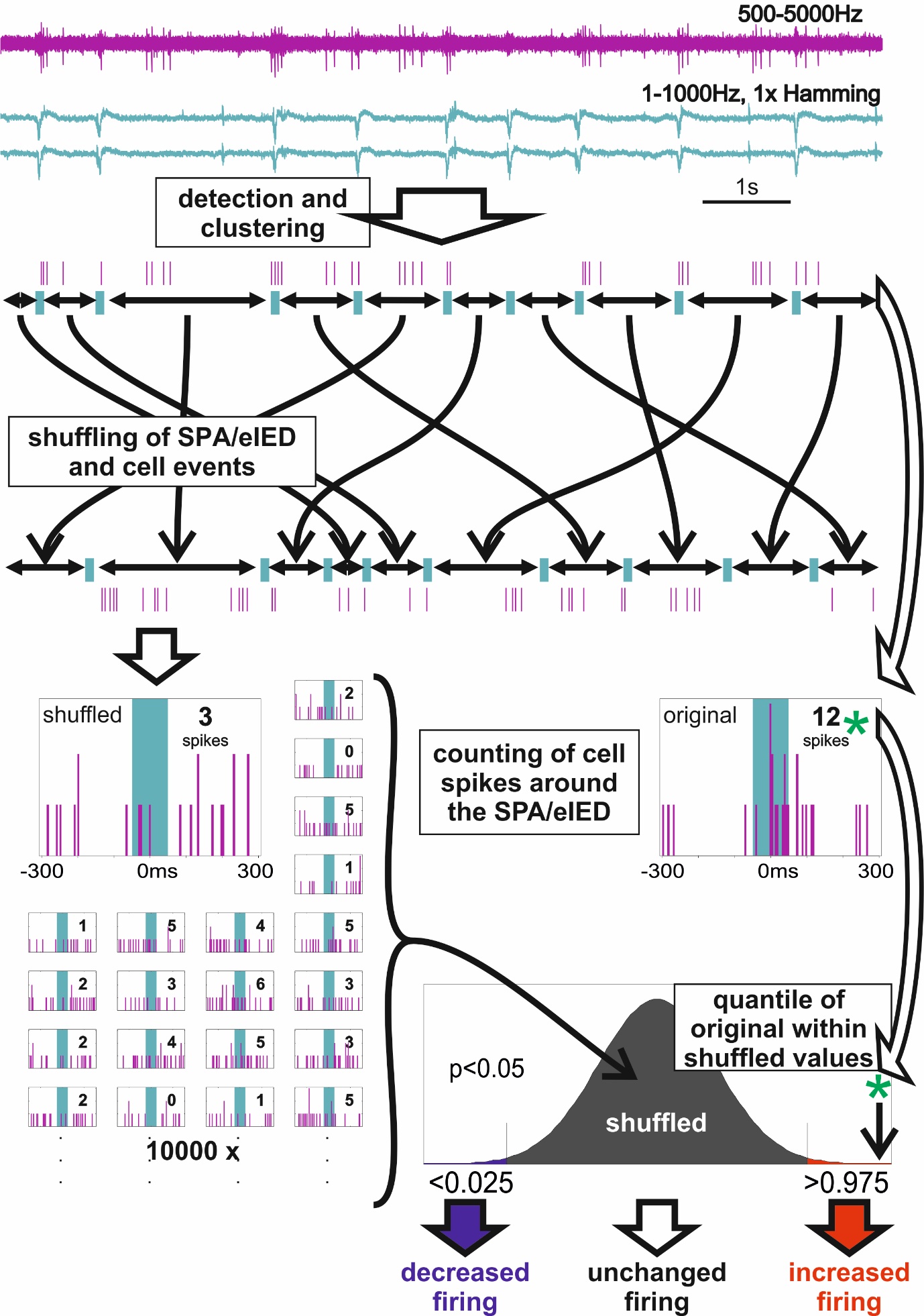


**Supplementary Figure 5. Calculation of firing change quantiles**

The original cell (purple) and SPA/eIED (turquoise) events were detected from the LFPg. Both were shuffled by random permutation of their ISIs. For the original as well as the shuffled event trains, the numbers of APs during the SPA/eIEDs ±50 ms were counted. This is illustrated by PETHs: purple bars which overlap the grey-blue background reflect APs which happened during the SPA/eIED. The randomised event trains provide a distribution of shuffled values, which the original value (green asterisk) is compared to. The resulting quantile determines the categorisation of the cell as decreasing, not changing or increasing its firing during the respective SPA/eIED.

Subsequently, the number of APs during the ±50 ms around the SPA/eIED event was counted for each (shuffled as well as original) pair of event trains. The shuffled event trains offer information about how many APs could be expected by chance for a cell and an SPA/eIED with the respective properties if there was no temporal relationship between them. The number of APs during the SPA/eIED derived from the original event train was then compared to the analogous values derived from 10,000 shuffled event trains of the same cell and SPA/eIED. The cell-SPA/eIED relationship was then quantified by calculating which quantile within the randomised values was equal to the original value. The resulting value ranges from 0 to 1. A value of e.g. 0.6 means that the original value is larger than (or equal to) 60% of the shuffled values and smaller than (or equal to) 40% of the shuffled values. As the quantile reflects how likely it is to attain the observed firing change value due to chance alone, they can be used to judge the significance of the firing change. Firing change quantiles up to 0.025 and above 0.975 were considered significant firing decreases or increases, respectively, at a 5% significance level. Note that the quantile calculated from the number of APs ±50ms around the SPA/eIED is identical to the quantile that could have been calculated from the cell firing frequencies during the SPA/eIED.

## Dependence on the number of SPA/eIED and cell events

This statistic is less sensitive to problems associated with a low number of detected SPA/eIED or cell events (resulting from low recurrence frequencies) than the firing change calculated without the randomisation step. The categorization into increased, unchanged and decreased firing is however not completely independent of the number of SPA/eIEDs or cells (Supplementary Fig. 6A1, A2 and A3). This is evident as the numbers of events significantly differ between increased, unchanged and decreased cells (number of SPA/eIED events: incr < decr: *P*<0.01, *ES*=0.66; unch < decr: *P*<0.01, *ES*=0.67; number of APs: unch < incr: *P*<1e-19, *ES*=0.68; incr < decr: *P*<0.01, *ES*=0.66; unch < decr: *P*<1e-07, *ES*=0.82). A low number of events thus tends to result in cells being identified as not significantly changing their firing rate rather than wrongly identifying them as decreasing their firing rate (see Supplementary Fig. 6B, especially the cases with low event numbers resulting in no APs during the SPA/eIED culminating in extreme reductions in firing rate which are identified as not significant). The statistics mentioned above also indicate that a high number of events seemed to be required for characterizing a cell as showing decreased firing. This can be explained as only cells with rather high baseline firing rates can show a significant decrease in firing during the SPA/eIED. However, a low number of detected events does not always result in unchanged firing and high event numbers do not guarantee a significant (i.e. increased or decreased) result, as shown by the high degree of overlap in the distributions shown in Supplementary Fig. 6A1, A2 and A3.


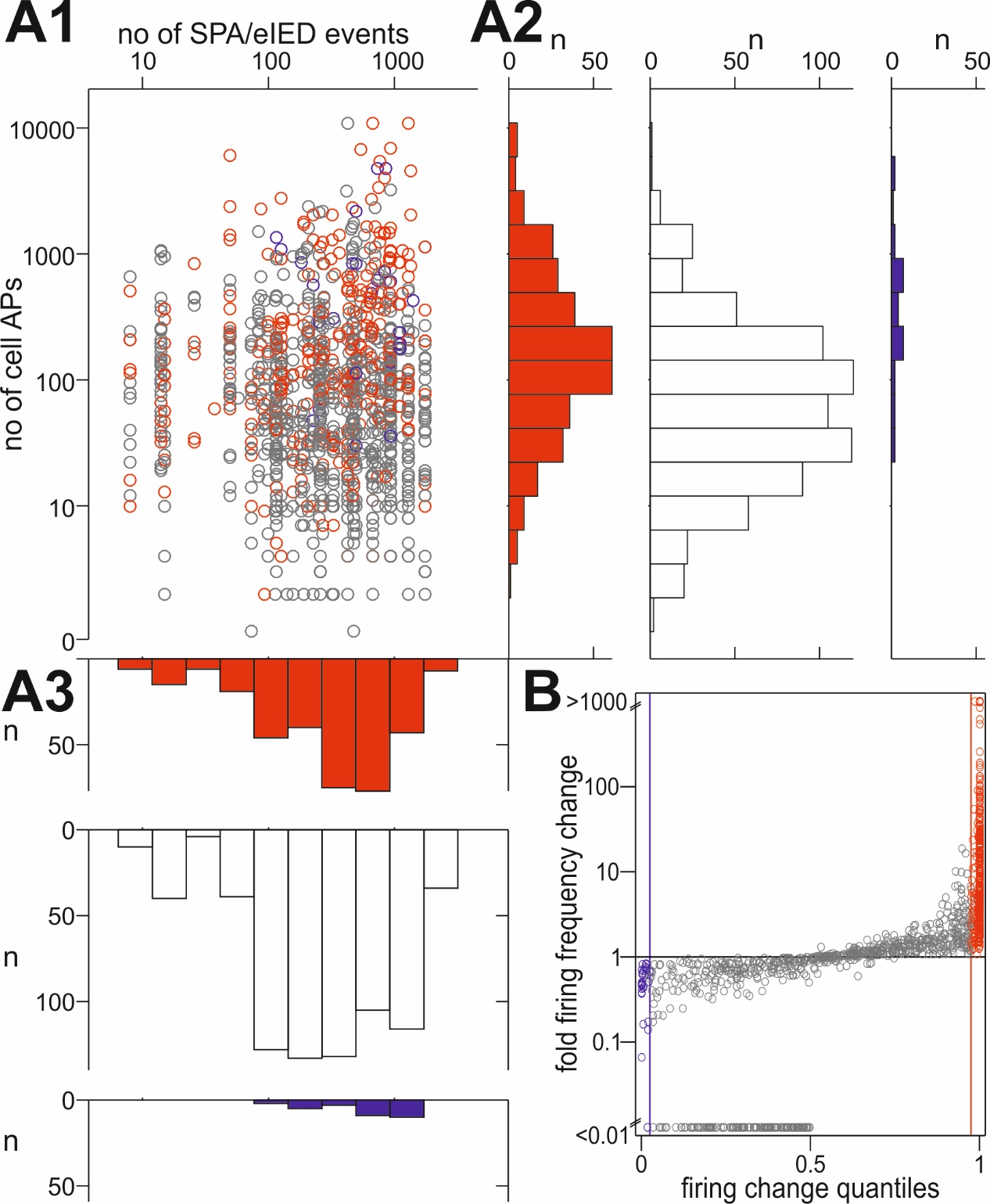


**Supplementary Figure 6. Properties of firing change quantiles**

A1, A2: Relationship between the number of cell events and increased (red), unchanged (white or grey) or decreased (blue) firing; A1, A3: Relationship between the number of SPA/eIED events and increased, unchanged or decreased firing; B: Relationship between the firing change quantiles and the ratio of cell firing frequency during the SPA/eIED to the firing frequency outside SPA/eIED.

## Dependence on the chosen time window

The choice of the time window used in the randomisation algorithm influences the resulting quantile and therefore the categorisation into increased, unchanged or decreased cell firing during the SPA/eIED. Thus, special care was taken in choosing an appropriate time window. Finally, a ±50 ms time window was adopted as it appeared to be best in fulfilling the following criteria:

1. High variance across quantiles compared to other time windows. High variance indicates that the quantiles can distinguish different patterns in the population, resulting in more increased and decreased patterns and fewer unchanged firing patterns (compare to Supplementary Fig. 7A).
2. Consistency of the chosen time window with time windows of similar length. This was quantified in various ways: The proportions of increased, unchanged and decreased cells do not change much compared to slightly wider or narrower time windows (flattened percentage curve in Supplementary Fig. 7A). Agreement was also quantified via the covariance matrix of the quantiles across time windows. Large covariance with quantiles of slightly wider or narrower time windows was deemed desirable. Moreover, the categorization disagreement was calculated for each pair of time windows. Disagreement means that the two time windows categorized the cell-SPA/eIED differently, e.g. unchanged according to one time window and increased or decreased according to the other time window. Out of the 1104 cell-SPA/eIED comparisons, there was only one case where two time windows disagreed to a more extreme extent: One time window (±5 ms, the smallest investigated time window) indicated increased firing, whereas the other time window (±80 ms, the longest investigated time window) indicated decreased firing. Visual inspection of the PETH in question showed that both categorizations were appropriate. The cell increased its firing very briefly followed by prolonged and strong decrease in firing. Supplementary Fig. 7B shows the disagreement of each pair of time windows calculated as the proportion of cases where the categorization differs between the two time windows.
3. The chosen time window should allow a compromise between detecting very brief (<10ms) and long-lasting (>200ms) changes in firing frequency.
4. The time window should be able to capture the firing frequency changes in the time period relevant to most cells (compare to Supplementary Fig. 7C)
5. The PETHs of cases of disagreement between similar time windows were visually assessed in order to choose the more appropriate time window.

**
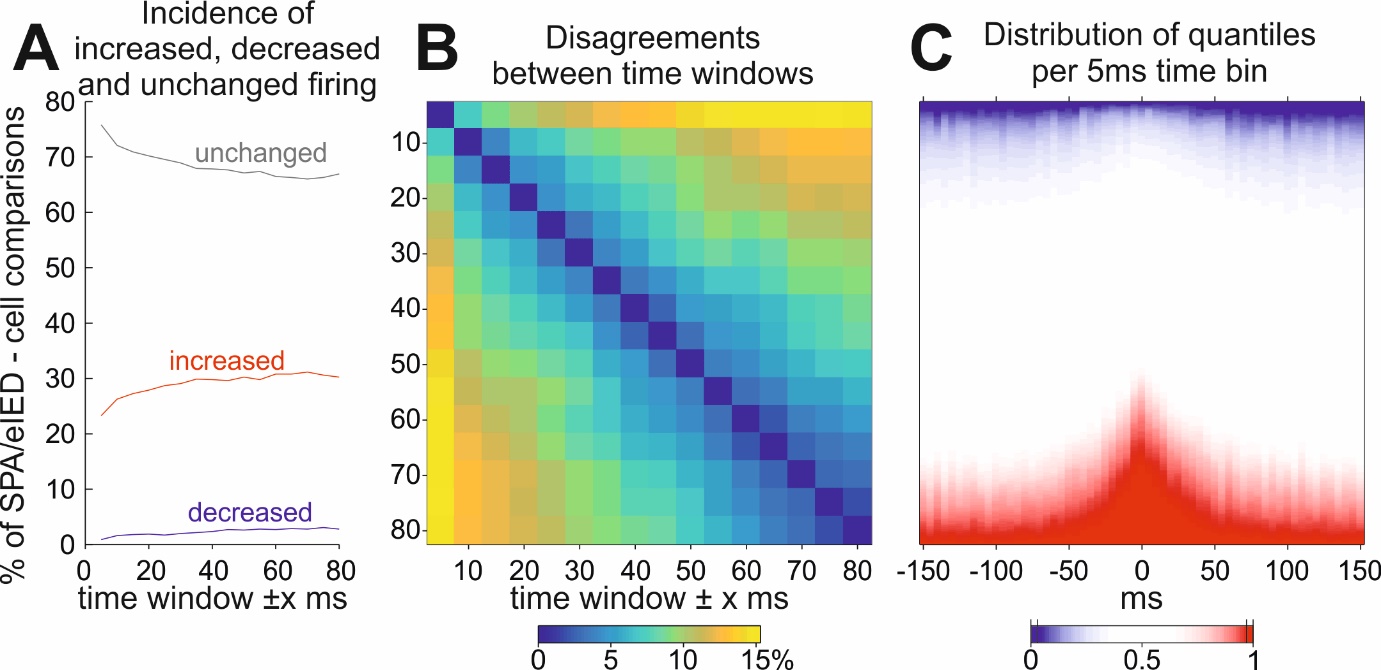
**

**Supplementary Figure 7. Effect of different time windows**

A: Proportion of cell-SPA/eIED comparisons which were categorized to display increased, unchanged or decreased firing; B: Disagreements (percent of non-matching categorizations) between pairs of time windows; C: Quantiles determined for each 5 ms bin for ±150 ms PETHs of all cell-SPA/eIED comparisons. Each time bin was then sorted by quantiles, showing a summary of the timing of all cell responses during SPA/eIEDs.

In addition to the quantile calculated for the time window of ±50 ms to determine whether a cell in- or decreased its firing, quantiles were also calculated for each of the 5 ms time bins in ±150 ms PETHs for visualisation (shown in Fig. 3 in the main text and Supplementary Fig. 7C). Moreover, quantiles were calculated for each of five relative phases.

## Relative time windows

The relative phases were defined in respect to the shape of the SPA/eIED LFPg transient. Determining whether the cell significantly in- or decreased its firing independently for each of the five relative phases allowed us to investigate the timing of different cell responses. In order to define these relative phases, the SPA/eIED width (duration) was measured on the channel displaying the largest LFPg amplitude, at 33% of that amplitude on the left (wl) and right (wr) side of the SPA/eIED peak. The phases were defined as multiples of these widths as shown in Supplementary Table 8 and illustrated in Supplementary Fig. 8).

**Supplementary Table 8. Definition of relative SPA/eIED phases**

Using the widths measured at 33% amplitude left (wl) and right (wr) of the peak. Compare to Supplementary Fig. 8.

| Phase | From | To |
| --- | --- | --- |
| Before | -4*wl | -2*wl |
| Ascending | -2*wl | -0.24*wl |
| Peak | -0.24*wl | 0.24*wr |
| Descending | 0.24*wr | 2*wr |
| After | 2*wr | 4*wr |


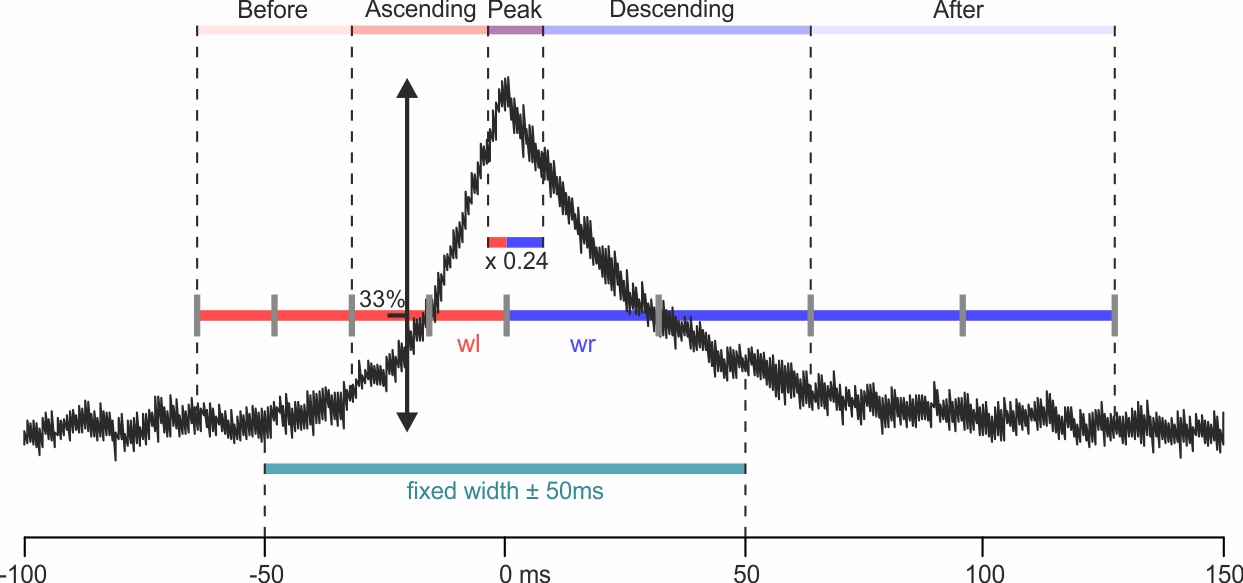


**Supplementary Figure 8. Illustration of relative SPA/eIED phases**

Illustration of the relative time windows as defined in Supplementary Table 3. Note that the ±50 ms time window (grey-blue) is independent of the shape of the SPA/eIED.

The increased, unchanged or decreased category was assigned to each phase for each cell-SPA/eIED based on the respective quantile. If a cell showed significantly increased firing only during one of the five phases, the cell was taken to increase its firing in that phase. If a cell showed significantly increased firing in multiple phases, out of those phases, the one with the highest firing frequency was chosen. If a cell did not show significantly increased responses during any of the five phases, no phase was assigned. This way, one or no relative phase was assigned to each cell-SPA/eIED case.

# Local vs. non-local neurons

Neurons were also categorized as local or non-local cells in relation to the SPA/eIED location. We found that higher percentages of local cells increased their firing rate (NoEpi SPA: 34.1%, ResEpi SPA: 59.1%, ResEpi eIED: 51.7%) than non-local neurons (NoEpi SPA: 12.3%, ResEpi SPA: 23.6%, ResEpi eIED: 13.0%) both in ResEpi and NoEpi tissue. The firing change quantiles were significantly higher for local cells (NoEpi SPA: 0.83 [0.42-1.00], ResEpi SPA: 1.00 [0.68-1.00]) compared to non-locals (NoEpi: 0.54 [0.30-0.85], *P*<10^-7^, *ES*=0.64; ResEpi: 0.73 [0.43-0.96], *P*<10^-8^, *ES*=0.69). The local vs. non-local difference was more pronounced during eIEDs (local: 0.98 [0.56-1.00], non-local: 0.49 [0.38-0.78], *P*<10^-5^, *ES*=0.75), mostly due to the lower participation of non-local cells during eIEDs than during SPAs (*P*<0.05, *ES*=0.61).

# Cell firing during multiple simultaneous SPA or SPA/eIED

We investigated cellular discharge during simultaneous multiple SPAs in slices from both ResEpi (n=57 cells) and NoEpi (n=158 neurons) patients, as well as in ResEpi slices exhibiting simultaneous SPA+eIED (n=48 neurons, Supplementary Fig. 9). In most of the cases two SPAs were generated in the same recording of the slice (123 cells in 7 recordings in NoEpi, 45 cells in 7 recordings in ResEpi), but we also detected triple simultaneous SPAs (37 cells in 4 recordings in NoEpi and 14 cells in 2 recordings in ResEpi). We differentiated cells with 1) no response to any of the emerging synchronies; 2) uniform responses, where the neuron’s firing rate either increased during all SPAs or decreased during all SPAs; and 3) differential responses, when the cell responded differently do different SPAs. Neurons with differential responses usually changed their firing rate during one or several, but not all SPAs. Only one cell showed an increased response during one as well as a decreased response during another SPA. This is not surprising as decreased responses are rare. Moreover, cells need a relatively high baseline firing rate to be identified as significantly decreasing their firing during an SPA, which limits the possibility of further increasing the firing rate during another SPA.

Expected numbers for ‘uniform’, ‘none’ and ‘differential’ responses were calculated from the percentages of increased, unchanged and decreased responses to SPAs. Supplementary Table 9 shows that ‘differential’ responses to multiple SPAs were most common in ResEpi tissue, while ‘none’ responses were most common in NoEpi tissue. However, this is to be expected as unchanged responses are much more common in NoEpi than in ResEpi. Overall, the case numbers we observed are comparable to those which would be expected. However, it seems that ‘none’ and ‘uniform’ responses are more common than expected, while ‘differential’ responses are less common than expected.

Comparable ratios were found during simultaneous SPA+eIEDs: 54.2% showed no response, 35.4% showed ‘differential’ responses and 10.4% showed ‘uniform’ responses. Eleven cells (22.9%) showed an increased firing rate during the SPA and unchanged during the eIED. Five neurons (10.4%) showed an elevated firing rate during the eIED but no response during the SPA, and one cell (2.1%) showed a decreased firing rate during the SPA and no change during the eIED.


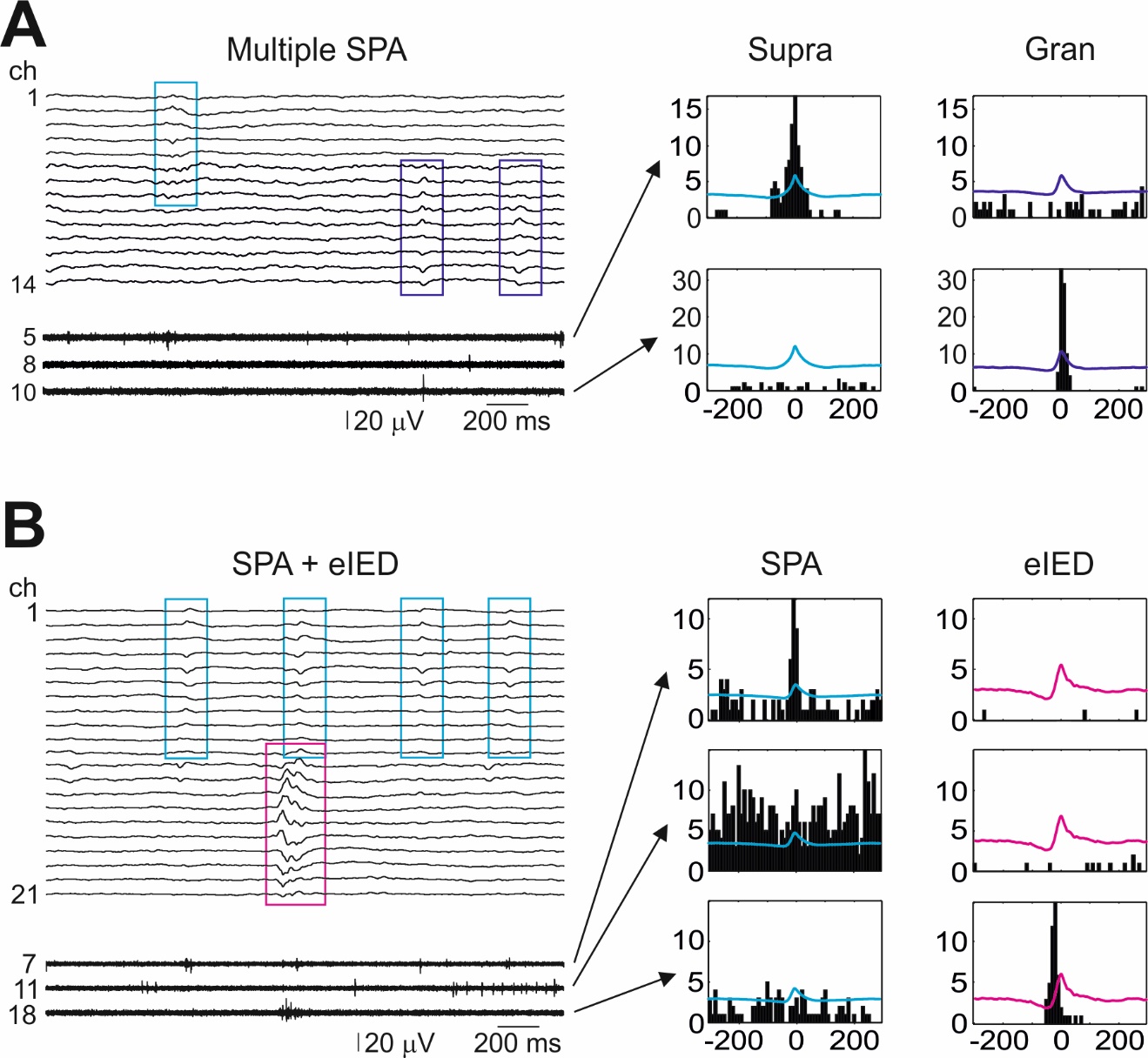


**Supplementary Figure 9. Cells during simultaneous multiple SPAs and/or eIEDs**

A) A considerable ratio of cells detected in recordings with multiple SPAs showed differential response to the synchronous activities, i.e. with increased firing to one of the SPAs, but no response to the other one. The firing of the cell on channel 5 is increased during supragranular SPA (light blue box on recording and light blue line on PETH) and remains unchanged during granular SPA (dark blue box and dark blue line). In contrast, the neuron on channel 10 shows increased firing during granular SPA and no response during supragranular SPA. Coloured lines on the PETHs show the LFPg average of the SPA/eIED

B) About one third of the cells showed differential response to simultaneous SPA+eIED activities. In this example, cell on channel 7 increased its firing rate during SPA (light blue box and light blue line) but not during eIED (magenta box and magenta line). The cell on channel 11 shows no response to any of the activities, whereas the neuron on channel 18 increases its firing rate during eIED but not during SPA.

**Supplementary Table 9. Cell responses during multiple SPAs**

Expected and observed numbers of ‘none’, ‘differential’ and ‘uniform’ responses during multiple SPAs. As 75.79% of NoEpi cells show unchanged responses, the number of expected ‘none’ responses (for 123 cells during 2 simultaneous SPAs and 37 cells during 3 simultaneous SPAs) can be calculated as 0.7579^2*123 + 0.7579^3*37 = 86.8. The number of uniform responses is the sum of only increased and only decreased responses. The differential responses encompass all remaining cases.

|  | Expected | | Observed | |
| --- | --- | --- | --- | --- |
|  | NoEpi | ResEpi | NoEpi | ResEpi |
| None | 86.8 (54.2%) | 15.4 (26.1%) | 91 (56.9%) | 23 (39.0%) |
| Differential | 67.0 (41.9%) | 34.6 (58.6%) | 57 (35.6%) | 25 (42.4%) |
| Uniform | 6.3 (3.9%) | 9.0 (15.3%) | 12 (7.5%) | 11 (18.6%) |
| Total | 160.0 | 59.0 | 160 | 59 |

We noticed that cells with differential responses usually increased their firing rate during the synchronous activity which was in their respective spatial vicinity (see also ‘Local vs. non-local neurons’ above). In NoEpi slices neurons increased their firing rate in 41 cases during local SPAs, and in 16 cases during distant activity. Cells showed unchanged firing to local events in 20 cases, and to distant SPA in 48 cases. In ResEpi slices, cells increased their firing rate to local SPAs in 16 cases, to distant SPAs in 7 cases. Cells showed no response to local events in 6 cases, and to distant activity in 20 cases. Decreased discharge rates to both local and distant SPA were found in 3 cases each in NoEpi and ResEpi. Note that the sum of these numbers is higher than the number of cells with differential responses, since we detected triple and quadruple activities as well. The same phenomenon was observed in case of simultaneous SPA+eIED. Eight cells increased their firing rate to local SPAs and 3 cells to distant SPAs showing no response to eIED. All five cells increasing their discharge rate to eIEDs with no change to SPA were cells local to the eIED. The cell showing a decrease in firing to the SPA and no response to eIED was local to the SPA.

# Hyperexcitability in ResEpi vs. NoEpi tissue

We observed certain differences in the cellular firing during SPA, when comparing slices derived from epileptic and non-epileptic patients. Along with a higher LFPg amplitude, a significantly larger pool of PCs and INs participated in the generation of SPAs in ResEpi tissue (higher proportion of cells with increased firing rate), together with a higher reliability during, and dependency on SPAs, than in NoEpi samples. These indicate that cells in the epileptic neocortex are under stronger control from the population and are more involved in the population activity than cells in non-epileptic tissue. Moreover, higher ratios of PC-PC interactions and PC-IN sequences were detected in ResEpi compared to NoEpi SPA (Supplementary Table 10). These results suggest that the finely tuned balance in the activation of excitatory and inhibitory microcircuits has been shifted towards an enhanced excitability and synchrony in epilepsy.


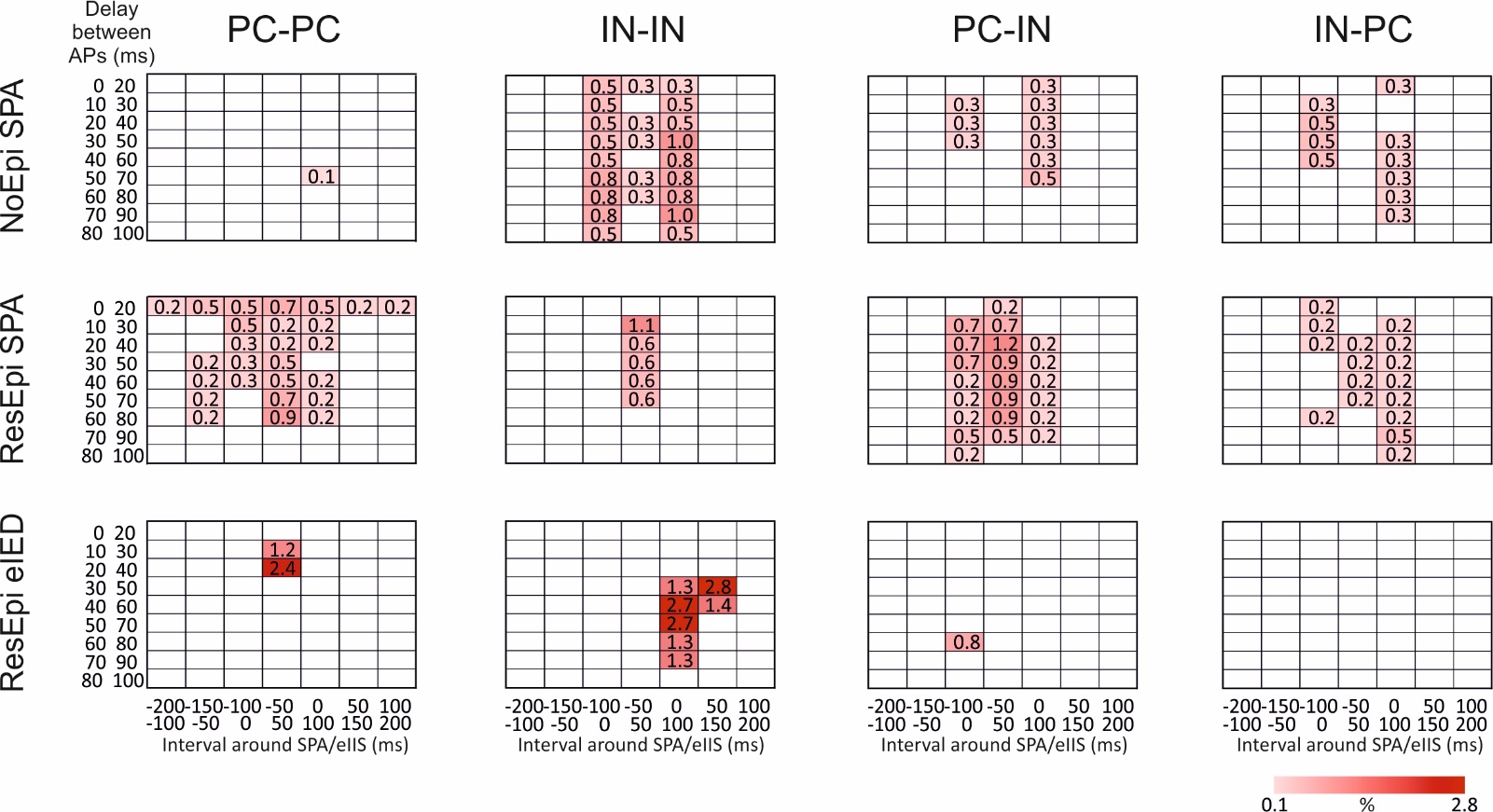


**Supplementary Table 10. Percentages of significant cell-cell interactions during SPA/eIED**

Percentages of significant cellular interactions are shown, checked at different, 100 ms long time intervals around the LFPg peak of the SPA/eIED events (between -200 and +200 ms, x-axes), as well as at different delays between the cells (20 ms long delay intervals, between 0 and 100 ms, y-axes).

The differences in the IN-IN interactions between SPAs and eIED was considerable (i.e., relatively low number of interactions, symmetrically around the peak of the SPA vs. higher numbers of interactions clearly following the peak of the eIED). This might result from several different phenomena. First, we have to emphasize, that these interactions were not monosynaptic connections, but were mediated through the neuronal network (see the time scale and the Methods section). One possible reason for the differences in the IN-IN interactions is, that the two different synchronous activities emerge in different neocortical laminae: SPA mainly in the supragranular, eIED in the infragranular layers. The very low number of direct PC-IN interactions during eIED might suggest that widely distributed sparse connections exist between individual PCs and INs transmitting low probability subthreshold excitatory potentials in the infragranular layers of the human neocortex, such as in the hippocampal CA3 region ^8,9^. The firing of the PCs at the earlier phases of eIEDs providing (bursting) excitatory input might sum up during eIED and induce the activation of INs. This scenario would contrast to the connectivity/cell activating pattern observed in the supragranular layers, where numerous PC-IN interactions have been detected during emerging SPA, and where single PCs can reliably initiate long synaptic sequences involving both PCs and INs ^10,11^. Another possible reason for the intense IN firing and high numbers of IN-IN interactions might be in relation with the depolarizing effect of GABA in epilepsy ^12^, which was described to affect pyramidal cells in the hippocampal formation ^13^. This phenomenon might take place in interneurons as well in the human neocortex, enhancing the probability of detecting more interactions between them. However, GABAergic input depolarizing postsynaptic interneurons remains to be demonstrated.

In summary, we think, that changes in the PC-PC, IN-IN, PC-IN interactions (but also the differences in the initiation of SPA vs. eIEDs) results from a combination of cellular and network characteristics, including layer specificity, presence of IB-PBs (in the infragranular layers), as well as of molecular, cellular and connectivity changes related to epilepsy.

# References

1. Kandrács A, Hofer KT, Tóth K*, et al.* Presence of synchrony-generating hubs in the human epileptic neocortex. *J Physiol* **597**, 5639-5670 (2019).

2. Tóth K, Hofer KT, Kandrács A*, et al.* Hyperexcitability of the network contributes to synchronization processes in the human epileptic neocortex. *J Physiol* **596**, 317-342 (2018).

3. Schevon CA, Ng SK, Cappell J*, et al.* Microphysiology of epileptiform activity in human neocortex. *J Clin Neurophysiol* **25**, 321-330 (2008).

4. van Breemen MS, Wilms EB, Vecht CJ. Epilepsy in patients with brain tumours: epidemiology, mechanisms, and management. *Lancet Neurol* **6**, 421-430 (2007).

5. Yli-Hankala A, Vakkuri A, Sarkela M, Lindgren L, Korttila K, Jantti V. Epileptiform electroencephalogram during mask induction of anesthesia with sevoflurane. *Anesthesiology* **91**, 1596-1603 (1999).

6. Stasiowski MJ, Marciniak R, Dulawa A, Krawczyk L, Jalowiecki P. Epileptiform EEG patterns during different techniques of induction of general anaesthesia with sevoflurane and propofol: a randomised trial. *Anaesthesiol Intensive Ther* **51**, 21-34 (2019).

7. Wang B, Bai Q, Jiao X, Wang E, White PF. Effect of sedative and hypnotic doses of propofol on the EEG activity of patients with or without a history of seizure disorders. *J Neurosurg Anesthesiol* **9**, 335-340 (1997).

8. Wittner L, Henze DA, Záborszky L, Buzsáki G. Three-dimensional reconstruction of the axon arbor of a CA3 pyramidal cell recorded and filled in vivo. *Brain Struct Funct* **212**, 75-83 (2007).

9. Le Duigou C, Simonnet J, Telenczuk MT, Fricker D, Miles R. Recurrent synapses and circuits in the CA3 region of the hippocampus: an associative network. *Frontiers in cellular neuroscience* **7**, 262 (2014).

10. Molnár G, Oláh S, Komlósi G*, et al.* Complex events initiated by individual spikes in the human cerebral cortex. *PLoS Biol* **6**, e222 (2008).

11. Szegedi V, Paizs M, Csákvári E*, et al.* Plasticity in Single Axon Glutamatergic Connection to GABAergic Interneurons Regulates Complex Events in the Human Neocortex. *PLoS Biol* **14**, e2000237 (2016).

12. Cohen I, Navarro V, Clémenceau S, Baulac M, Miles R. On the origin of interictal activity in human temporal lobe epilepsy in vitro. *Science* **298**, 1418-1421 (2002).

13. Szabadics J, Varga C, Molnár G, Oláh S, Barzó P, Tamás G. Excitatory effect of GABAergic axo-axonic cells in cortical microcircuits. *Science* **311**, 233-235 (2006).
